# A modified phase-retrieval algorithm to facilitate automatic *de novo* macromolecular structure determination in single-wavelength anomalous diffraction

**DOI:** 10.1107/S2052252524004846

**Published:** 2024-06-21

**Authors:** Xingke Fu, Zhi Geng, Zhichao Jiao, Wei Ding

**Affiliations:** a Beijing National Laboratory for Condensed Matter Physics, Institute of Physics Chinese Academy of Sciences Beijing100190 People’s Republic of China; b Beijing Synchrotron Radiation Facility, Institute of High Energy Physics Chinese Academy of Sciences Beijing100049 People’s Republic of China; c School of Physical Sciences University of Chinese Academy of Sciences Beijing 100049 People’s Republic of China; d Songshan Lake Materials Laboratory, Dongguan523808, People’s Republic of China; Harima Institute, Japan

**Keywords:** substructure determination, single-wavelength anomalous diffraction, SAD, phase-retrieval algorithm, tangent formula, macromolecular crystallography, automatic *de novo* structure determination

## Abstract

The success of experimental phasing in macromolecular crystallography relies primarily on the accurate locations of heavy atoms bound to the target crystal. To improve the process of substructure determination, a modified phase-retrieval algorithm built on the framework of the relaxed alternating averaged reflection (RAAR) algorithm has been developed. Importantly, the proposed algorithm features a combination of the π-half phase perturbation for weak reflections and enforces the direct-method-based tangent formula for strong reflections in reciprocal space. The proposed algorithm is extensively demonstrated on a total of 100 single-wavelength anomalous diffraction (SAD) experimental datasets, comprising both protein and nucleic acid structures of different qualities. Compared with the standard RAAR algorithm, the modified phase-retrieval algorithm exhibits significantly improved effectiveness and accuracy in SAD substructure determination, highlighting the importance of additional constraints for algorithmic performance. Furthermore, the proposed algorithm can be performed without human intervention under most conditions owing to the self-adaptive property of the input parameters, thus making it convenient to be integrated into the structural determination pipeline. In conjunction with the *IPCAS* software suite, we demonstrated experimentally that automatic *de novo* structure determination is possible on the basis of our proposed algorithm.

## Abstract 2

A modified phase-retrieval algorithm has been built on the framework of the relaxed alternating averaged reflection (RAAR) algorithm, incorporating the π-half phase perturbation for weak reflections and the direct-methods based tangent formula for strong reflections in reciprocal space. The modified phase-retrieval algorithm exhibits significantly improved effectiveness and accuracy of various forms of SAD substructure determination to facilitate automatic *de novo* macromolecular structure determination.

## 1. Introduction

Despite recent advances in cryo-electron microscopy and artificial intelligence-based structure predictions, X-ray crystallography still plays an important role in unraveling protein structural details at the atomic level. Owing to significant advancements in synchrotron technology (Chapman, 2023 ▸) and continuous developments of novel methodologies, there has been a substantial increase in the number of crystal structures deposited in the Protein Data Bank (PDB) over the past two decades (Berman *et al.*, 2000 ▸). One of the well known crystallographic structural determination techniques is experimental phasing, which remains a unique way to solve novel protein structures without known homologues (Hendrickson, 2023 ▸). Moreover, experimental phasing is commonly adopted to determine crystal structures of nucleic acids due to a lack of sufficient structural diversity for molecular replacement (Zhang *et al.*, 2020 ▸; Schneider *et al.*, 2023 ▸). In addition, in the presence of radiation-induced severe site-specific damage of heavy-atom derivatives in microcrystal electron diffraction (Micro-ED) (Martynowycz *et al.*, 2020 ▸; Hattne *et al.*, 2018 ▸), or in some other challenging cases (Bunkóczi *et al.*, 2015 ▸; El Omari *et al.*, 2023 ▸), experimental phasing is still indispensable for structural determination.

The general method of choice for experimental phasing is single-wavelength anomalous diffraction (SAD) (Rose & Wang, 2016 ▸), which requires data collection at a wavelength in proximity to the absorption edge of a chosen anomalous scatterer. Depending on the type of anomalous scatterers, the SAD technique can be categorized into several variations, such as Se-SAD (labeling proteins with seleno­methio­nine), M-SAD (natural metalloproteins), *X*-SAD (artificially introduced iodine, bromine or other metal ions) and native-SAD (intrinsic sulfur, phospho­rus or other light atoms, and other ions inherently or inadvertently introduced). By fully exploiting the weak anomalous difference signals between Bijvoet pairs of acentric reflections, the heavy atoms attached to the target crystal (referred to as the substructure) can be accurately identified, which in turn provide initial phase information for further structural determination.

However, the quality of diffraction data can fluctuate significantly for different crystals, thus necessitating the development of diverse approaches for SAD substructure determination. Hitherto, there have been three mainstream methods to solve heavy-atom substructures in SAD. The first method is based on the Patterson function, which can be generally categorized into vector-search methods (Knight, 2000 ▸; Hu *et al.*, 2019 ▸) and superposition methods (Buerger, 1959 ▸; Sheldrick, 1998 ▸; Grosse-Kunstleve & Brunger, 1999 ▸; Terwilliger & Berendzen, 1999 ▸; Burla *et al.*, 2007 ▸). The second method involves the tangent formula-based direct methods, which are capable of solving the phase problem using only the intensity information (Karle & Hauptman, 1956 ▸). By incorporating direct methods into a dual-space iteration framework (Fan *et al.*, 2014 ▸), which involves applying the tangent formula in reciprocal space while enforcing the atomicity constraint in real space, the effectiveness and accuracy of heavy-atom substructure solution have been remarkably improved. This strategy has been adopted by the most widely used SAD substructure determination software suites, such as *SHELXD* (Schneider & Sheldrick, 2002 ▸) and *HySS* (Grosse-Kunstleve & Adams, 2003 ▸). The third potential method is represented by the *ab initio* phase-retrieval algorithms (Liu *et al.*, 2012 ▸; Palatinus, 2013 ▸; Skubák, 2018 ▸), which can also recover the phase information from diffraction intensities alone by iterative application of constraints in both spaces. However, unlike the direct methods based dual-space strategy, the phase-retrieval algorithms simply impose experimental moduli constraints in reciprocal space and require no compositional information.

In chemical crystallography, one of the most widely used phase-retrieval techniques is the charge flipping (CF) algorithm (Oszlányi & Sütő, 2004 ▸), which simply reverses the signs of a proportion of lowest-density values in direct space. Despite its extreme simplicity, researchers are increasingly seeking to enhance the performance of the CF algorithm. In 2005, the convergence property of the CF algorithm is significantly leveraged by introducing the π-half phase perturbation to the weak reflections (that is, the phases of a percentage of weakest reflections are shifted by a constant of π/2) (Oszlányi & Sütő, 2005 ▸). In addition, combined with the tangent formula (Coelho, 2007*a* ▸) or histogram matching (Baerlocher *et al.*, 2007 ▸), the CF algorithm can also be used to determine small-molecule crystal structures that are difficult to solve. Benefiting from its outstanding performance and the development of a series of user-friendly computer programs, like *SUPERFLIP* (Palatinus & Chapuis, 2007 ▸) and *TOPAS* (Coelho, 2007*b* ▸), the CF algorithm is further extended to macromolecular crystallography, including directly solving macromolecular structures (Dumas & Lee, 2008 ▸; Coelho, 2021 ▸) as well as SAD substructure determination (Dumas & Lee, 2008 ▸). However, the success rate of the CF algorithm when applied to macromolecular crystallography is relatively low, being heavily dependent on the data quality, and it requires substantial iterations for convergence, thus hindering its wide applications. In order to improve the performance of phase-retrieval algorithms in SAD substructure determination, the relaxed averaged alternating reflection (RAAR) algorithm (Luke, 2005 ▸) is implemented specifically in a crystallographic context, which outperforms the CF algorithm in terms of SAD substructure determination (Skubák, 2018 ▸). However, it remains unclear whether the improvements that have been made in the CF algorithm can also be applied to the RAAR algorithm and achieve superior performance in SAD substructure determination.

Based on the current progress, we proposed a modified phase-retrieval algorithm built on the framework of the RAAR algorithm which synergistically combines the π-half phase perturbation for weak reflections while simultaneously enforcing the tangent formula for strong reflections with sufficiently high-intensity values in reciprocal space to facilitate SAD substructure determination. In order to validate the general applicability of our proposed algorithm, a total of 100 sets of SAD experimental data of different quality were used for study. Importantly, the proposed algorithm could successfully determine most of the heavy-atom substructures with a success rate of more than 90%, demonstrating the remarkable robustness and versatility of our algorithm. Compared with the standard RAAR algorithm, the proposed algorithm brought about a higher success rate and achieved better heavy-atom coordinate precision. Finally, the modified phase-retrieval algorithm for solving heavy-atom substructures was integrated into the structure solution pipeline *IPCAS* (*Iterative Protein Crystal structure Automatic Solution*) (Ding *et al.*, 2020 ▸) to enable the automation of *de novo* macromolecular structure determination.

## 2. Methods

### 2.1. Theoretical background

In this section, some theoretical foundations behind the modified phase-retrieval algorithm are summarized as follows. First, to provide a comprehensive understanding, we begin by introducing the fundamental principles of SAD phasing. Subsequently, a general description of the phase-retrieval algorithms is presented. In addition, the classical CF algorithm as well as some of its important variants that will be adopted in this study are shown. Finally, a brief introduction to the RAAR algorithm is provided.

In the SAD experiment, due to the anomalous scattering of heavy atoms, the reflections **F**(*hkl*) and **F**(−*h*−*k*−*l*) will have different intensities and their phases are no longer complementary. Let the amplitudes of **F**(*hkl*) and **F**(−*h*−*k*−*l*) be denoted 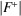 and 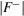; hence. the relationship between the Bijvoet difference Δ*F*^±^, the phase of the protein φ_T_ and that of the anomalous substructure φ_A_ can be expressed as
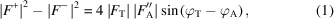
Here, 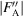 is the imaginary component of *F*_A_ (Hendrickson, 1979 ▸). If the contribution of the anomalous scattering to the total diffracting power of the crystal is small, 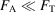 and 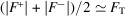 (Hendrickson *et al.*, 1985 ▸), then
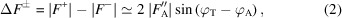
if 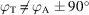. The phase ambiguity of the phase of the protein φ_T_ can be express as (Ramachandran & Raman, 1956 ▸)
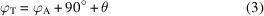
or
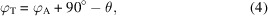
where 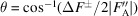. The methods for breaking the phase ambiguity have been summarized in some reviews (Dauter *et al.*, 2002 ▸; Rose & Wang, 2016 ▸; Hendrickson, 2023 ▸). Therefore, the solution of an anomalous substructure is crucial for subsequent macromolecular structure determination.

The phase-retrieval algorithms belong to a type of perturbation-based dual-space iterative algorithm, which aims to find a harmonious balance between real and reciprocal space. This iterative process can be mathematically expressed as
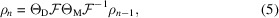
where ρ_*n*_ is the electron-density map calculated at the *n*th iteration; 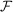 and 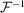 denote the forward and inverse Fourier transforms; and Θ_M_ and Θ_D_ correspond to the constraint operators in reciprocal and real space, respectively. In general, the measured structure-factor magnitudes impose a stringent constraint on experimental data consistency in reciprocal space. In real space, due to the atomicity nature, a majority of values in the crystal unit cell are close to zero and the structure information is only confined within a small region [see Figure 1 in Oszlányi & Sütő (2008 ▸)].

For the standard CF algorithm, the experimental amplitude constraint and low-density perturbation are iteratively employed to explore the parameter space. Specifically, in reciprocal space, the calculated Fourier amplitudes (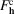) will be replaced by those observed (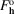) while keeping the phases and the unobserved Fourier amplitudes unchanged:
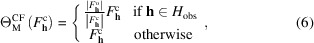
where **h** represents the Miller indices and *H*_obs_ is the set of experimentally measured reflections. In real space, the signs of electron densities that are lower than a specified threshold are flipped, while others are kept unchanged:
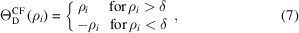
where δ signifies the threshold of electron-density values, which can affect the quality of the recovered map.

In order to improve the performance of the CF algorithm, several variants have been designed by introducing different perturbations into the dual space (Palatinus, 2013 ▸). For example, one noticeable improvement of the CF algorithm is the use of π-half phase perturbation for weak reflections in reciprocal space, where the calculated phases for observed weak reflections are modified according to the following formula:
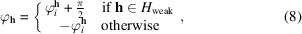
where 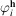 denotes the calculated phases at the current iteration and *H*_weak_ is the set of weak reflections. It has been extensively demonstrated that such a modification can dramatically improve the performance of the CF algorithm. Another improvement of the operation on the calculated phases in reciprocal space is the integration of the tangent formula into the CF algorithm (Coelho, 2007*a* ▸). Specifically, after inverse Fourier transform of the real-space constraint electron-density map, the calculated phases for a percentage of observed strong reflections are further modified according to the following equations:
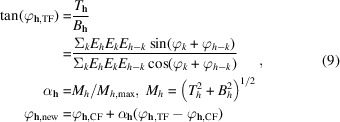
where 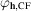 represents the calculated phases produced by the CF algorithm at each iteration, *T*_**h**_ and *B*_**h**_ denote the numerator and the denominator of the tangent formular, *E_h_* is the normalized structure factor, *M_h_* is a reliability factor determining the confidence level of the tangent formula-generated phases 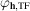, 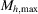 is the maximum value across all selected strong reflections and 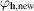 is the modified phases. Note that instead of directly replacing the calculated phases with the tangent formula-generated phases, a scale factor α_**h**_ is adopted to compensate for the inaccuracy of the tangent formula, where a higher value will give more weight to the tangent formula-generated phases and *vice versa*. It is also worth highlighting that the requirement of the positivity constraint in real space should be lifted under poor-resolution conditions, where the absolute values for density are taken (Coelho, 2007*a* ▸). In addition, the zero Fourier coefficient *F*(0) deserves special attention, which can never be measured experimentally. In most cases, its value is allowed to fluctuate freely during iterations. However, it is sometimes useful to constrain *F*(0) to zero throughout the calculation (Palatinus, 2004 ▸; Coelho, 2007*a* ▸; Zhou & Harris, 2008 ▸).

In terms of SAD substructure determination, the RAAR algorithm has recently emerged as a superior alternative to the CF algorithm (Skubák, 2018 ▸). Strikingly, it can enlarge the radius of convergence and improve the success rate in solving heavy-atom substructures. The basic RAAR algorithm can be written as
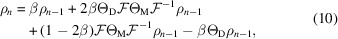
where β is a coefficient of the relaxation term, the reciprocal-space constraint operator Θ_M_ is essentially the same as equation (6) and the real-space constraint operator Θ_D_ is expressed as follows:
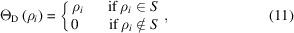
where *S* indicates the support where the object is located (Luke, 2005 ▸; Martin *et al.*, 2012 ▸). In SAD substructure determination, since the support of heavy atoms cannot be determined, the judgment criteria in equation (7) is therefore applied to the RAAR algorithm, but slightly modified to take into account the last calculated density map in our study. After simplification of equations (10) and (11), the real-space constraint of our modified RAAR algorithm can be conveniently expressed as
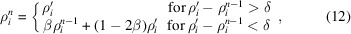
where δ signifies the threshold of electron-density values, 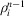 denotes the calculated density map at last iteration and 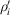 represents the current density map updated by the reciprocal-space constraint.

The phase problem in crystallography is an inconsistent problem. Compared with other phase-retrieval algorithms such as the low-density elimination (LDE) algorithm (Shiono & Woolfson, 1992 ▸), CF algorithm, hybrid input–output (HIO) algorithm (Fienup, 1982 ▸) and averaged alternating reflections (AAR) algorithm (Bauschke *et al.*, 2004 ▸; Oszlányi & Sütő, 2011 ▸), the RAAR algorithm tends to exhibit a superior ability to escape local minima and avoid divergence (Palatinus, 2013 ▸). Luke (2005 ▸) demonstrated that the HIO algorithm is highly parameter-dependent for different data. In contrast, the RAAR algorithm offers a simpler and mathematically tractable approach that outperforms other phase-retrieval algorithms. Therefore, the RAAR algorithm presents a promising alternative for solving the crystallographic phase problem, yet it remains understudied within the crystallography context.

### 2.2. The workflow of the modified phase-retrieval algorithm

**Figure 1:**
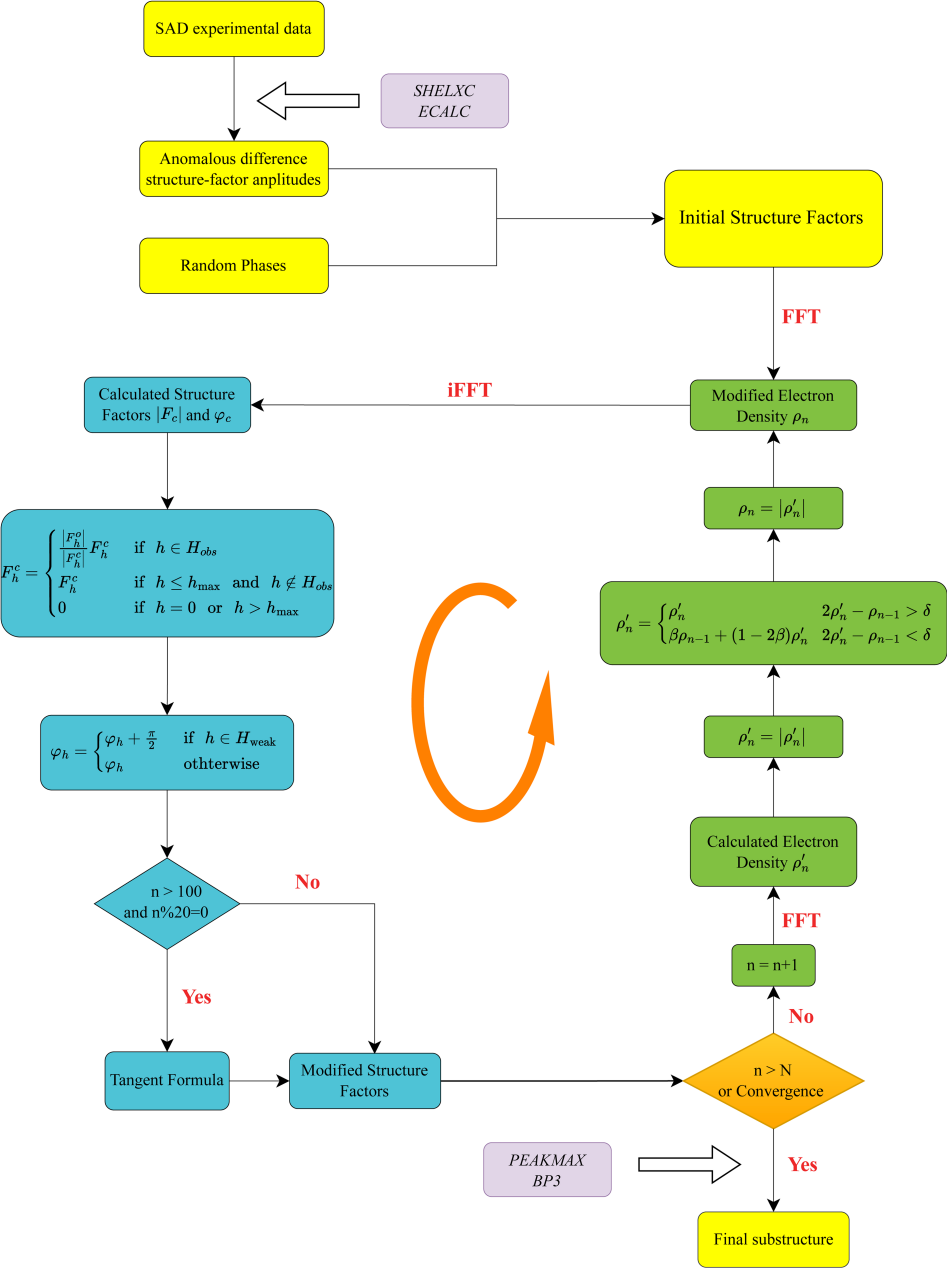
Schematic flowchart of the modified phase-retrieval algorithm. The different stages are highlighted with different colors: the yellow segment signifies the initialization of the algorithm, involving the generation of anomalous difference amplitudes, normalization, and the construction of the initial electron density with a combination of random phases and normalized anomalous amplitudes; the blue segment encompasses reciprocal-space constraints, such as amplitude constraint, π-half phase perturbation for weak reflections and the tangent formula; the green segment represents the direct-space constraints, including the standard RAAR algorithm and positivity constraint. Several third-party programs used for data preparation, heavy-atom peak location and substructure refinement are highlighted in pink. *n* and *N* represent the number of the current iteration and the predefined maximum iteration number, respectively.

Based on the above theoretical foundations, a modified dual-space iterative algorithm is proposed for SAD substructure determination in this section. The modified phase-retrieval algorithm is built on the basic RAAR algorithm and incorporates a number of important improvements that have been made in the CF algorithm as mentioned above. A flowchart of the modified phase-retrieval algorithm is presented in Fig. 1 ▸ and the detailed iterative process is described as follows:

(*a*) Initially, a random electron-density map (ρ_0_) placed in the crystal unit cell is generated from the symmetry-expanded observed anomalous difference structure factors combined with random phases satisfying Friedel’s law. Of note, all unobserved anomalous difference structure factors are set to 0 in this step.

(*b*) The real electron density is inverse Fourier transformed to obtain the calculated structure factors, |*F*_c_| and φ_c_, within the whole reciprocal space, which are further reduced to the asymmetric unit according to Laue symmetry. To this end, crystallographic symmetry information will be enforced in reciprocal space.

(*c*) Replace the calculated structure factor moduli with measured moduli while retaining the calculated phases [see equation (6)]. Three types of reflections are distinguished here: (i) observed reflections, which are directly replaced by measured moduli; (ii) unobserved reflections within the resolution limit, which are allowed to change freely; and (iii) high-frequency reflections beyond the resolution limit, which are forced to be zero. In addition, some unobserved reflections that are systematically extinct are also forced to be zero. Special attention should be paid to the zero Fourier coefficient *F*(0), which is set to zero throughout the calculation.

(*d*) Modify the calculated phases by means of π-half phase perturbation and tangent formula. Specifically, the phases are firstly shifted by 90° for a certain fraction of observed reflections that are considered to be weak at each iteration according to equation (8). Afterwards, the phases for a specified number of strongest reflections are further refined based on the tangent formula according to equation (9). Note that the tangent formula-based constraint is applied every 20 iterations, instead of at each iteration, after 100 cycles of the iterative process to compensate for the excessive phase perturbations.

(*e*) A new set of symmetry-expanded calculated structure factors subtending the whole reciprocal space are synthesized and converted to a new density 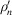 via Fourier transform.

(*f*) Density modification is applied to 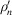 on the basis of the RAAR algorithm according to equation (12). Note that the absolute values of 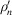 are taken both before and after density modification to enhance the positivity constraint in real space.

(*g*) The modified density is transformed back to calculated structure factors via inverse Fourier transform and steps (*b*)–(*f*) are repeated until convergence or a predefined iteration number is reached.

In order to monitor the convergence of the phase recovery procedure, we tried three different figures of merit for comparison, including the classical crystallographic *R* factor, electron-density skewness (Terwilliger *et al.*, 2009 ▸) and the standard Pearson correlation coefficient (CC). We observed that the Pearson CC can best distinguish between successful and unsuccessful SAD substructure determination (for more details, refer to Section S1 of the supporting information). As a result, the Pearson CC is used to evaluate the iterative process of the above-mentioned algorithm. The Pearson CC between *E*_o_ and *E*_c_ is shown below,
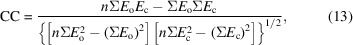
where *E*_o_ and *E*_c_ represent the observed and calculated normalized amplitudes, respectively; *n* represents the number of observed reflections; and *E*_c_ is derived from the Fourier transform of the electron-density map after real-space restraints.

In the modified phase-retrieval algorithm, there are some parameters that need to be carefully adjusted, including the relaxation parameter β, the electron-density threshold δ, the percentage of weak reflections *w*_best_ and the number of strong reflections *N*_TF_. In our algorithm, δ is dynamically adjusted to keep a fixed proportion of low-density values that will be perturbed. Through numerous trials, it is empirically found that a constant value of 0.82 for β and a percentage of 13% for δ are most suitable for algorithmic performance. In addition, it is computationally observed that the percentage of weak reflections *w*_best_ is better kept within the range 20–50%. In practice, the optimal value of *w*_best_ varies significantly for different experimental datasets and is therefore automatically determined in the proposed algorithm (for more details, refer to Section S2 of the supporting information). For the number of strong reflections *N*_TF_, we simply follow the rules as stated below. When the number of total observed reflections is lower than 5000, *N*_TF_ is set to 1000. When the number is above 5000 but below 8000, *N*_TF_ is set to 1300. When the number is above 8000, *N*_TF_ is increased to 1500.

### 2.3. Implementation of the modified phase-retrieval algorithm for SAD substructure determination

In SAD substructure determination, the first step is to accurately extract the anomalous difference structure factors *F*_A_ from the observed diffraction data. According to equation (1), the structure factors of anomalous atoms from the diffraction intensity data contain the information from non-anomalous atoms. However, according to equation (2), it can be derived that
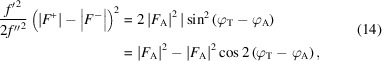
where 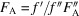. The second term in equation (14) represents the noise term since φ_T_ and φ_A_ are uncorrelated. Therefore, the amplitudes of *F*_A_ can be expressed as the absolute difference between reflections of Bijvoet pairs, 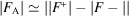, calculated using the *SHELXC* program in this study (Sheldrick, 2008 ▸), which rejects a large number of reflections according to the statistical characteristic of diffraction intensity. The rejection can improve the quality of anomalous difference structure factors. As the normalized structure factors are required for the tangent formula, the calculated anomalous difference structure factor amplitudes are further normalized for SAD substructure determination using the *ECALC* program from the *CCP4* suite (Collaborative, 1994 ▸). Moreover, the success in applying phase-retrieval algorithms to substructure determination depends somewhat on the high-resolution truncation of reflections since the anomalous signal typically extends to lower than the overall data resolution. Additionally, high-resolution anomalous signals are always corrupted with numerous noises, thus making substructure determination very sensitive to the high-resolution cutoff parameter. A simple scheme to determine the high-resolution cutoff value is to truncate the anomalous data to a level about 0.5 Å lower than the diffraction maximum (Sheldrick, 2008 ▸; Usón & Sheldrick, 2018 ▸). In addition, 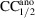 (Karplus & Diederichs, 2012 ▸) at a cutoff value of 0.3 serves as another good indicator, and CC_range_ (Skubák, 2018 ▸), a combination of multiple resolution cutoffs, is sometimes used to find the optimal high-resolution cutoff. In this study, the ratio of the anomalous difference to its standard deviation (|Δ*F*|/σ(Δ*F*) = 1.2) (Usón & Sheldrick, 2018 ▸) is adopted as the criterion to estimate the anomalous resolution.

Once the anomalous difference data with a reasonable resolution are ready, the next important step is to implement the modified phase-retrieval algorithm as mentioned above to solve heavy-atom substructures. Since phase-retrieval algorithms start with random phases, not every calculation can converge successfully. In practice, it is possible to perform several attempts initiated with different random phases and pick the best one with the highest CC value. For each unknown structure, a total of 400 trials with different random phases are performed and each trial consists of 500 or 750 Fourier iterations.

From the best reconstructed electron-density map, a peak search procedure will be carried out to determine the 3D coordinates of all potential heavy-atom substructures in the asymmetric unit. In this study, the *PEAKMAX* program from the *CCP4* suite is adopted for this purpose, which can output a list of peaks ordered by the height of the density peaks. Afterwards, the potential heavy atoms are chosen from these sorted peaks based on a user-defined cutoff number, which is two greater than the number of deposited heavy atoms. Moreover, note that heavy-atom refinement against the experimental data can, under most circumstances, further improve the accuracy of substructure atoms. As an optional procedure, the *BP3* program (Pannu *et al.*, 2003 ▸) from the *CCP4* suite is used in this work to refine the 3D atomic coordinates, occupancy and temperature factor for each potential heavy atom. Ultimately, the calculated heavy atoms are utilized to deduce initial phases for structure determination, which are further refined through multiple rounds of density modification and model building. In our study, the *IPCAS* structure solution pipeline is applied to automate the entire structure determination process, with the calculated heavy atoms serving as the sole input information.

In order to quantitatively measure the success of a substructure determination, the calculated substructure atoms are compared with the actual heavy atoms extracted from the reference PDB coordinates based on the *SITCOM* program (Dall’Antonia & Schneider, 2006 ▸), which can output the match rate and corresponding positional difference. In our study, the SAD substructure determination is considered to be successful when more than 50% of the heavy-atom sites can be correctly matched to the reference substructure. For the purpose of comparison, the fraction of heavy-atom sites that are correctly identified as well as their root mean square deviations (r.m.s.d.s) of positional difference are adopted as the main indicators to evaluate the quality of SAD substructure determination.

### 2.4. Test data

A total of 100 SAD experimental datasets, consisting of both protein and nucleic acid structures of different data quality, were randomly downloaded from the PDB using advanced search with the structure determination method matching to SAD to test the modified phase-retrieval algorithm. The test data provide a wide range in terms of resolution (spanning from 1.1 to 3.9 Å) and space group, covering all seven crystal systems and anomalous scatterers. In summary, there are 55 sets of Se-SAD, 16 sets of S-SAD and 29 sets of *X*-SAD. The complete list of these PDB entries with detailed information are given in Section S5 of the supporting information. All calculations presented in this paper were performed on a Dell computer with Intel(R) Xeon(R) Gold 5222 at 3.80 GHz, 8-core Inter Xeon W CPU, 64 GB RAM.

## 3. Results

### 3.1. Experimental validation of the modified phase-retrieval algorithm

In order to provide an evaluation of the power of the modified phase-retrieval algorithm in SAD substructure determination, a typical SAD experimental dataset (PDB entry 6e9c; Zhou *et al.*, 2019 ▸) containing a total of 15 Se atoms in the asymmetric unit was used as an example for detailed algorithmic analysis. For the purpose of comparison, the standard CF algorithm, the standard RAAR algorithms without π-half phase perturbation and tangent formula constraint, or with only π-half phase perturbation were also performed. Of note, all four algorithms were initiated with the same random phase values and run with identical parameters for 750 Fourier iterations to ensure an objective comparison.

**Figure 2:**
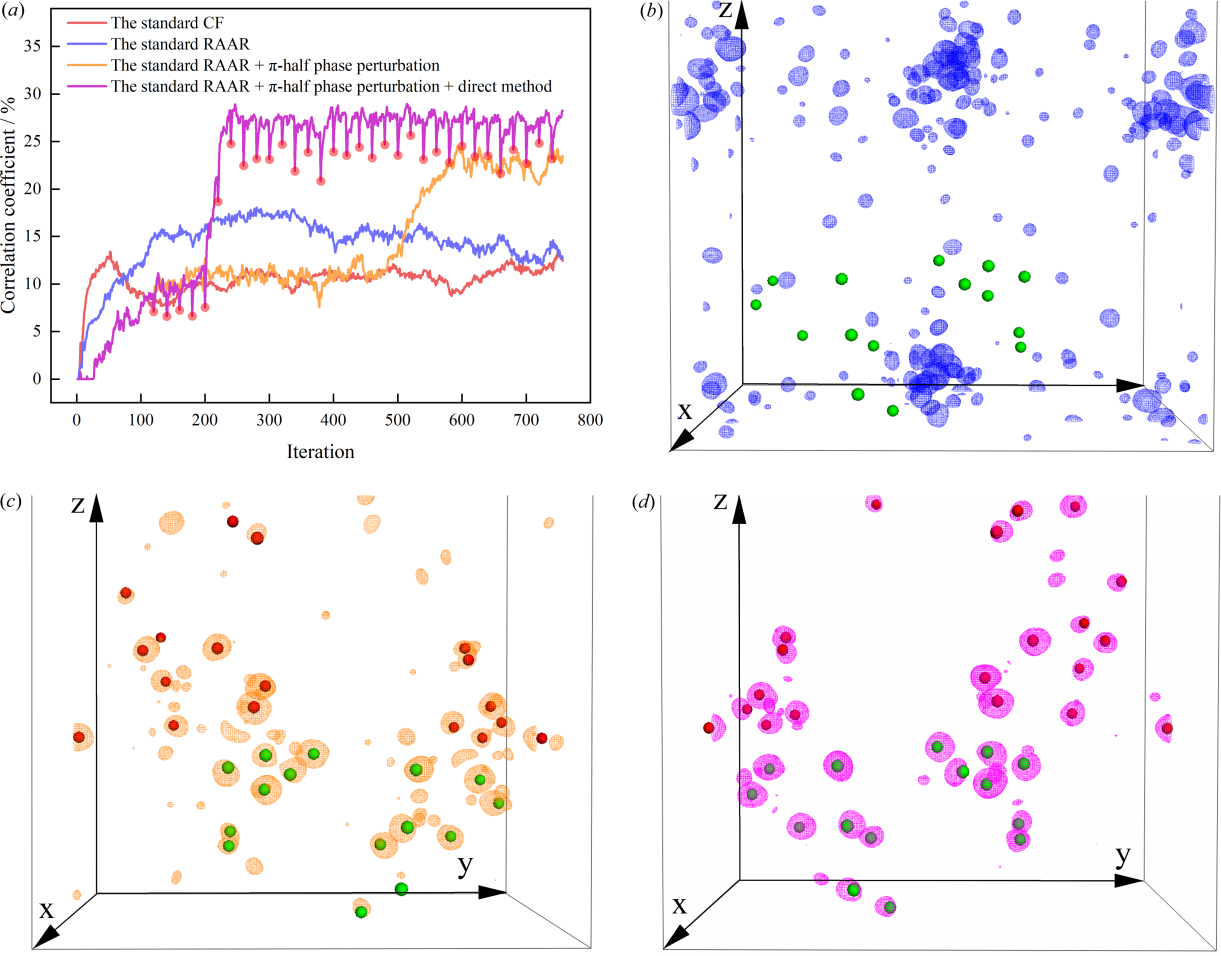
Comparison of different substructure determination algorithms for a protein using PDB entry 6e9c. (*a*) The runs of four different phase-retrieval algorithms with and without phase constraints (the π-half variant and tangent formula) across 750 Fourier iterations, all starting with the same random phase values. The red dots represent the use of the tangent formula. (*b*)–(*d*) Recovered electron-density maps of the three different RAAR algorithms superimposed with the reference heavy atoms. The standard RAAR algorithm is shown in blue [(*a*) and (*b*)], the standard RAAR algorithm incorporating π-half phase perturbation for weak reflections is shown in orange [(*a*) and (*c*)], and the standard RAAR algorithm incorporating the π-half phase perturbation and tangent formula (*i.e.* the modified phase-retrieval algorithm) is shown in purple [(*a*) and (*d*)]. The green balls represent 15 Se atoms in the asymmetric unit from the PDB-deposited structure and the red balls are the equivalent Se sites that are symmetry expanded according to the space-group information. The directions of the three unit-cell axes are also shown in the maps and all three electron-density maps are contoured at the same value of 5σ.

The evolution of CC values as a function of iterations for the four algorithms are compared in Fig. 2 ▸(*a*), revealing significantly different converging trends. Obviously, it can be observed that the CC of the standard CF algorithm as well as the standard RAAR algorithm only converge to a value of ∼15%, much lower than that of the other two algorithms, both of which are higher than ∼25%. This demonstrates that the π-half phase perturbations for weak reflections can help the RAAR algorithm overcome stagnation and converge towards the correct solution. Note that an additional application of the tangent formula for strong reflections further increases the CC value from ∼25 to ∼30%, suggesting the potential of tangent formula to facilitate phase recovery. In chemical crystallography, a dramatic change of certain quality metrics, such as the *R* factor or CC, is generally indicative of the successful convergence of the iterative phase retrieval procedure. In our study, we did not observe a sharp increase in the standard CF and RAAR algorithms even reaching 2000 iterations, meaning the standard CF and RAAR algorithms are likely to fail in substructure solution. On the contrary, there is an abrupt increase in the CC at the ∼500th iteration after applying the π-half phase perturbation to the standard RAAR algorithm, and this number is reduced to ∼200 on further application of the tangent formula constraint. The above observation indicates that the π-half phase perturbation can expand the phase space to increase the convergence radius, while the tangent formula constraint can significantly accelerate convergence. Of particular note, the tangent formula constraint would result in a decrease in CC, as indicated by the in red dots in Fig. 2 ▸(*a*). One possible reason is that the tangent formula introduces a significant perturbation, which will disrupt the temporary balance between the real and reciprocal spaces. However, such perturbation is sufficient to help the algorithm escape from its stagnation at local minima.

The recovered electron-density maps with the reference substructure superimposed for the three different RAAR algorithms are presented in Figs. 2 ▸(*b*)–2 ▸(*d*). In addition, the potential heavy atoms sites were extracted from the map using the *PEAKMAX* program and compared with the reference substructure using the *SITCOM* program. Apparently, the electron-density map calculated from the standard RAAR algorithm could hardly coincide with the reference substructure [Fig. 2 ▸(*b*)], and no potential heavy atom sites could be matched to the reference substructure. In contrast, a more interpretable electron-density map is obtained after incorporating the π-half phase perturbation into the standard RAAR algorithm [Fig. 2 ▸(*c*)]. Note that the handedness of substructures can hardly be solved by the phase-retrieval algorithm alone due to its inherent randomness. As a result, the recovered electron-density map may sometimes be centrosymmetric to the final accurate substructure, as depicted in Fig. 2 ▸(*c*). However, after substructure alignment using the *csymmatch* program from the *CCP4* suite, most of the aligned reference heavy atoms, with the exception of only one, could be accurately mapped onto this electron-density map. As expected, based on the *SITCOM* analysis, 14 out of 15 Se atoms could be correctly identified from the potential heavy atom sites, consistent with the above observation. After integrating both the π-half phase perturbation and the tangent formula constraint within the RAAR algorithm, all 15 heavy atoms could be correctly identified from the resulting high-quality map [Fig. 2 ▸(*d*)] and well matched with the reference substructure. Nevertheless, there are still some noise peaks present in the recovered density maps, and the lowest peak height of the correctly identified heavy atoms is used to characterize the noise level. The lowest peak height is estimated to be 7.07× the standard deviation (7.07σ) of the recovered map when applying only π-half phase perturbation, whereas this increases to 9.49σ when further enforcing the tangent formula constraint. Taken together, it is experimentally demonstrated that the modified phase-retrieval algorithm exhibits significantly enhanced efficiency and accuracy for SAD substructure determination in comparison with the standard RAAR algorithm.

### 3.2. General applicability of the modified phase-retrieval algorithm

**Figure 3:**
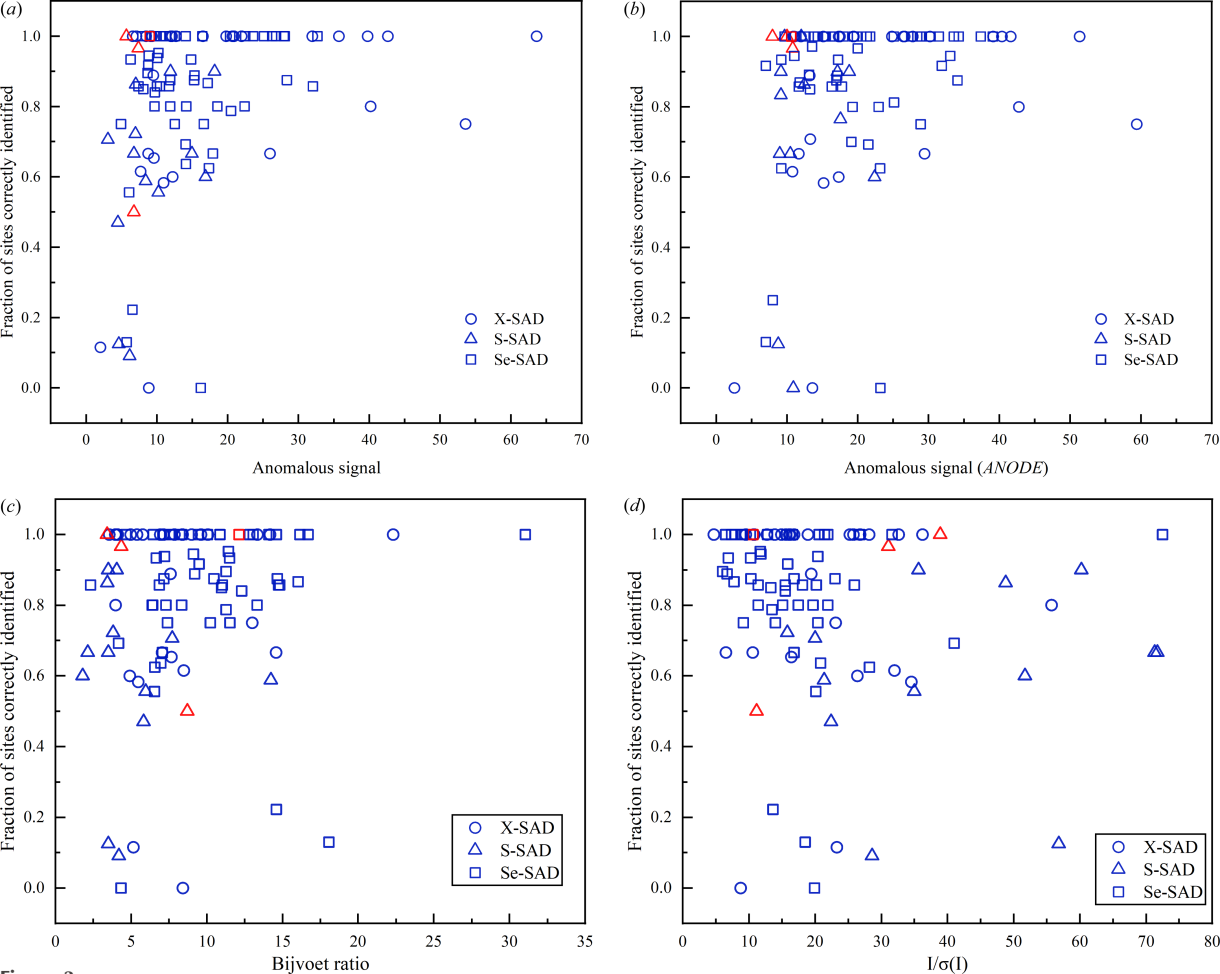
Evaluation of the success rate of substructure determination against the anomalous signal, Bijvoet ratio and SNR using 100 SAD datasets. (*a*) Fraction of heavy-atom sites correctly identified as a function of the anomalous signal calculated with the first method. (*b*) Fraction of sites correctly identified as a function of the anomalous signal calculated with the second method. (*c*) Fraction of sites correctly identified plotted against the Bijvoet ratio (in units of percentage). (*d*) Fraction of sites correctly identified plotted against the SNR. Note that the anomalous signal is in units of σ, which is the standard deviation of the anomalous difference electron-density map. Each symbol in the graph represents a single dateset. The circle, triangle and square represent the *X*-SAD dataset (*X* represents iodine, bromine or metal ions), S-SAD dataset and Se-SAD dataset, respectively. The substructure searches carried out with default parameters are shown in blue and the red ones indicate substructures failed to be determined initially but that could be solved by further adjustment of some parameters.

In order to demonstrate the generality of the modified phase-retrieval algorithm for SAD substructure determination, a total of 100 SAD experimental datasets were used for a comprehensive analysis. Without loss of generality, the same procedure was carried out on each test case with all necessary parameters automatically determined. Fig. 3 ▸(*a*) shows the fraction of correctly identified heavy atoms for all 100 SAD datasets, which are further classified according to the type of scatterers. In total, there were 89 datasets that could be automatically processed to yield correct heavy atoms with a match rate of more than 50%. For the other 11 datasets, an additional 4 datasets, marked in red in Fig. 3 ▸(*a*), could be successfully processed after fine-tuning some of the parameters, such as high-resolution cutoff, *w*_best_ and *N*_TF_. For the remaining 7 SAD datasets that were unsuccessfully processed using the modified phase-retrieval algorithm, a further test was implemented using the *SHELXD* program with the same high-resolution cutoff for 10 000 trials. However, there was still no solution to these 7 datasets. Although we cannot exclude the possibility that some substructures could be determined by further adjustment of certain parameters, it can still be concluded that the modified phase-retrieval algorithm is on par with the traditional best substructure determination method.

In order to explore the reason behind the failure of some SAD datasets, the anomalous signal, the type of scatterers, the Bijvoet ratio, the signal-to-noise ratio (SNR) together with the truncated anomalous resolution were analyzed for each dataset. In this study, we adopted two separate approaches to estimate the anomalous signal of each dataset for comparison. First, the anomalous signal is estimated by averaging the peak height at the reference heavy-atom sites in the anomalous difference Fourier map (Bunkóczi *et al.*, 2015 ▸; Terwilliger *et al.*, 2016 ▸), which is calculated by combining the anomalous difference magnitudes from *SHELC* with the accurate phases derived from the PDB structure using the *FFT* program from the *CCP4* suite (Collaborative, 1994 ▸). Second, the anomalous difference Fourier map is calculated with *ANODE* (Thorn & Sheldrick, 2011 ▸) using the final refined models as the phase source; and the peak heights from this difference map are used for the estimation of the anomalous signal strength in the coordinates of anomalous scatters from the PDB structure. Of note, the calculation of the fraction of correctly identified heavy atoms is different for the two methods. In the first method, the identified heavy atom sites are directly compared with the reference substructure extracted from the PDB model. In the second method, the potential heavy atoms are compared with a list of strongest unique anomalous peaks from anomalous difference Fourier map generated with *ANODE*. Comparisons of the fraction of the correct substructure against the anomalous signal for both methods are shown in Fig. 3 ▸(*a*) and Fig. 3 ▸(*b*), respectively. It can be observed that the strength of anomalous signal calculated from *ANODE* [Fig. 3 ▸(*b*)] is slightly higher than that of the first method [Fig. 3 ▸(*a*)], which is attributed to the different programs used to calculate the anomalous difference map. In addition, the fraction of correct sites for the second method [Fig. 3 ▸(*b*)] is somewhat higher than that of the first method [Fig. 3 ▸(*a*)]. This is because the number of strong anomalous peaks is sometimes fewer than the final reference substructure as there may be unmodelled anomalous scatterers. Nevertheless, both methods tend to exhibit a highly similar overall distribution between the success rate of substructure determination and anomalous signal. From Figs. 3 ▸(*a*) and 3 ▸(*b*), it can be speculated that the success of substructure determination is not dependent on the specific type of scatterers, as there is no clear distinction for each class of scatterers in terms of the fraction of correctly identified heavy atoms, even for the most challenging S-SAD datasets. However, as shown in the bottom left corner in Figs. 3 ▸(*a*) and 3 ▸(*b*), the anomalous signals for all 7 failed datasets are mostly less than 10σ, which is generally considered to be weak (Terwilliger *et al.*, 2016 ▸), suggesting that the success of substructure determination may be largely affected by the strength of anomalous signal. Furthermore, the Bijvoet ratio [Fig. 3 ▸(*c*)] and SNR [Fig. 3 ▸(*d*)] are also analyzed for each dataset. It can be observed that most failed datasets show a tendency to have a smaller Bijvoet ratio and lower SNR. However, the success of substructure determination is much less dependent on either the Bijvoet ratio or SNR compared with the anomalous signal. The Bijvoet ratio is useful for acquiring a general idea about how large the anomalous signal is, but some errors in measurement may substantially affect the anomalous signal, thus making it less effective to measure the success of substructure determination. It is further demonstrated that no obvious correlation could be made between the anomalous signal and SNR of the diffraction data, which is shown by a relatively low Pearson CC [Fig. S5(*a*)]. This can be explained by the fact that the strength of the anomalous signal largely depends on the scattering ability and number of heavy atoms rather than the SNR of diffraction data. As shown in Fig. S5(*b*), all 7 failed datasets are truncated within a normal resolution range between 2 and 4 Å, suggesting that the truncated anomalous resolution has negligible influence on the success rate of substructure determination.

As mentioned above, the success of substructure determination is very likely to be dependent on the strength of the anomalous signal. Nevertheless, there are still some SAD datasets with anomalous signals below 10σ that could be successfully determined [33 out of 40 datasets in Fig. 3 ▸(*a*) or 9 out of 13 datasets in Fig. 3 ▸(*b*)]. For example, two SAD datasets with the PDB entries 6s1d (Nass *et al.*, 2020 ▸) and 6fms (Huang *et al.*, 2018 ▸) exhibit weak anomalous signals of 7.92 and 7.0σ, respectively, whose anomalous peak heights from the anomalous difference Fourier map generated with *ANODE* are listed in Table 1 ▸. For the 6s1d dataset, there are a total of 9 anomalous peaks from native sulfurs, all of which can be accurately matched with the identified heavy atom sites. For the 6fms dataset, there are a total of 12 anomalous peaks originating from selenium atoms, 11 of which can be accurately matched with the potentially solved substructures. The only misaligned selenium site comes from the last anomalous peak whose height is as low as 4.03σ. With this in mind, the modified phase-retrieval algorithm can be exceptionally powerful for SAD substructure determination in some challenging cases with weak anomalous signals.

**Figure 4:**
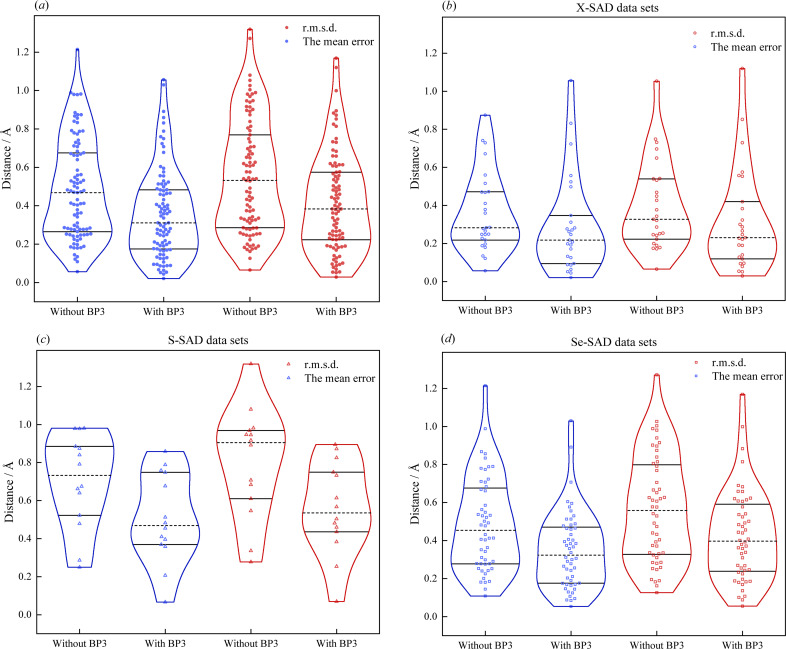
Comparison of the distribution of positional difference of correctly identified heavy atoms from the reference substructures before and after refinement with *BP3*. (*a*) Distribution of positional differences for all 93 SAD datasets. (*b*) Distribution of positional differences for only the *X*-SAD datasets. (*c*) Distribution of positional differences for only the S-SAD datasets. (*d*) Distribution of positional differences for only the Se-SAD datasets. Both the mean error (in blue) and the r.m.s.d. (in red) are used to evaluate the positional difference. Note that each dot represents a dataset and the horizontal width of the distribution reflects the frequency of each dataset falling within this range. The three lines in each group from up to down indicate the 75th percentile value, median value and 25th percentile value, respectively.

To quantitatively evaluate the accuracy of substructure determination, the mean and the standard deviation of the positional difference of correctly identified heavy atoms against the reference substructures were calculated for all 93 successful datasets [Fig. 4 ▸(*a*)]. Obviously, a majority of the substructures are determined with the mean positional difference less than 1.0 Å and the median value is 0.431 Å, indicating highly accurate substructure determination. Likewise, the standard deviation of the positional difference shows a similar distribution but with a somewhat larger median value. To further improve the accuracy of substructures, heavy-atom refinement against the experimental anomalous data was carried out using the *BP3* program. The mean and standard deviation of the positional difference after refinement are also presented in Fig. 4 ▸(*a*) for comparison. Apparently, the positional difference of the refined substructures is significantly reduced, with a much lower median value of 0.29 Å, reflecting the effectiveness of heavy-atom refinement. However, note there are still some datasets showing increased positional difference after heavy-atom refinement, probably due to the poor quality of these experimental data. The positional difference in terms of different types of anomalous scatters are also analyzed and the observation for each type of scatterer generally holds the same as above [Figs. 4 ▸(*b*)–4 ▸(*d*)]. Note that the most significant improvement in substructure refinement is made in the case of S-SAD datasets, possibly because the initial positional difference is remarkably higher than the others. In addition, it is also observed that some datasets with relatively large positional differences are always concomitant with low resolution. To this end, the relationship between positional difference and truncated anomalous resolution was analyzed, where datasets with lower anomalous resolution tend to bring about increased positional uncertainty of heavy-atom substructures (for more details, refer to Section S3 of the supporting information).

**Figure 5:**
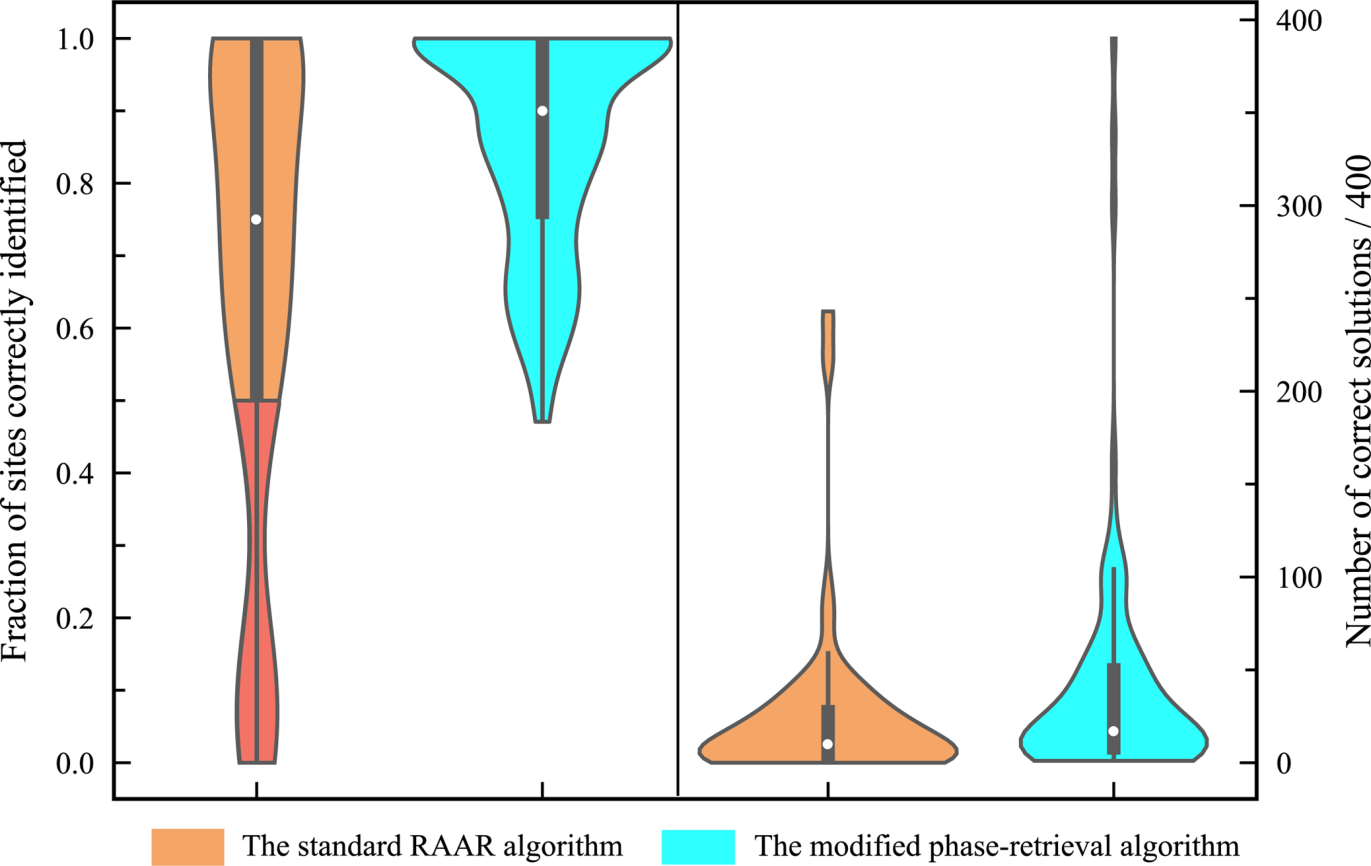
Comparison of the standard RAAR algorithm (in orange) and the modified phase-retrieval algorithm (in cyan) for the 93 SAD datasets successfully solved by the modified phase-retrieval algorithm. The left panel of the graph indicates the fraction of sites correctly identified using both algorithms, and the red area indicate unsolved substructures. The right panel indicates the number of the 400 trials that converged to correct solution for each dataset.

For the purpose of comparison, the standard RAAR algorithm without applying either π-half phase perturbation or the tangent formula constraint was also carried out on the same 100 SAD datasets with the same parameters for substructure determination. In contrast to the modified phase-retrieval algorithm, only 72 datasets, excluding the 7 failed ones mentioned above, could be successfully processed using the standard RAAR algorithm. The fraction of correctly identified heavy atoms, as well as the success rate expressed as the number of successful convergences out of 400 trials, are comparatively illustrated for both the standard RAAR algorithm and the modified phase-retrieval algorithm in Fig. 5 ▸ (for more details, refer to Section S4 of the supporting information). It can be seen that there are more substructures that could be solved with higher completeness and success rate when employing the modified phase-retrieval algorithm. This demonstrates that the modified phase-retrieval algorithm in general outperforms the standard RAAR algorithm for SAD substructure determination.

On the whole, the test results on the 100 SAD datasets confirm that incorporating additional phase constraints in reciprocal space can significantly enhance the convergence radius of the algorithm and improve not only the accuracy but also the success rate for SAD substructure determination. In addition, the modified phase-retrieval algorithm is capable of dealing with the most challenging native-SAD datasets and can be conveniently integrated into other structure determination pipelines owing to the self-adaptive characteristic of the input parameters.

### 3.3. Automatic structure determination based on the modified phase-retrieval algorithm

**Figure 6:**
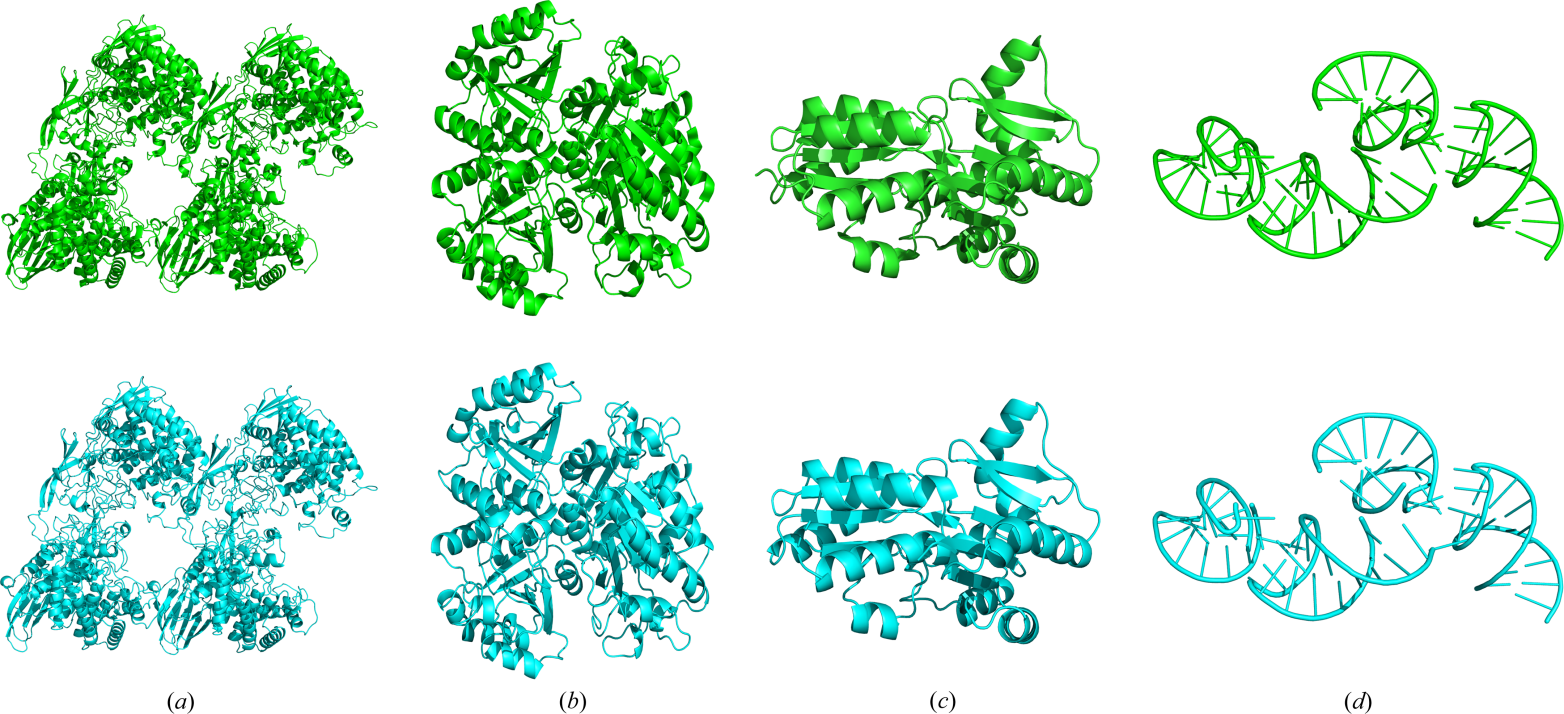
Cartoon representation of the four typical macromolecular structures automatically determined by the *IPCAS* pipeline using the heavy-atom substructure from the proposed algorithm as input (drawn in cyan). The corresponding models deposited in PDB (drawn in green) are also shown for comparison. (*a*) Representative structure with PDB entry 4qk0. (*b*) Representative structure with PDB entry 3s2s. (*c*) Representative structure with PDB entry 3fys. (*d*) Representative structure with PDB entry 5ndi.

Based on the substructures determined with the modified phase-retrieval algorithm, automatic structure determination was further carried out using the *IPCAS* software. *IPCAS* is a direct methods based pipeline for automatic protein structure determination. Within the framework of *IPCAS*, initial phases are determined by breaking the phase ambiguity in SAD experimental phasing via *OASIS* (Hao *et al.*, 2000 ▸), followed by multiple rounds of phase improvement, model building and structure refinement. The input information to *IPCAS* includes a list of heavy atoms with the occupancy and temperature factor, amino acid sequence, and diffraction data. In this study, the heavy atoms determined using the modified phase-retrieval algorithm from four representative examples [PDB entries 4qk0 (Lansky *et al.*, 2014 ▸), 3s2s (Liu *et al.*, 2011 ▸), 3fys (Nan *et al.*, 2009 ▸) and 5ndi (Huang *et al.*, 2017 ▸)] were input into *IPCAS* for automatic structure determination. The quality of each output model is evaluated based on the figure of merit (FOM), r.m.s.d., *R*_work_/*R*_free_, model completeness and model accuracy. Completeness is calculated by counting the proportion of auto-built residues in the sequence of the deposited PDB structure. Accuracy is calculated by counting the proportion of residues built correctly (a correctly built residue is one that is at a distance of at most 2 Å from a true Cα position in the deposited PDB structure). The results of the structure determination for these four representative cases are listed in Table 2 ▸ and structure comparisons between the calculated models and deposited PDB models after alignment are shown in Fig. 6 ▸. The time for each cycle of the proposed phase-retrieval algorithm to solve the substructure and the time for each cycle of *IPCAS* for automatic structure determination are also listed in Table 2 ▸.

As shown in Table 2 ▸, for the four test cases, the FOMs are all above 0.35, suggesting the reliability of phase values calculated with the positions of identified anomalous scatterers. In addition, the deviations of the automatically determined structures from the reference PDB models (r.m.s.d) are all below 0.3 Å, indicating highly accurate automatic structure determination. For the three protein structures, both the *R*_work_ and the *R*_free_ values fall below 0.26, and their completeness and accuracy both exceed 96%, This result is further confirmed by a careful examination of each calculated protein structure, which shares a sufficiently high structural similarity to the PDB model [Figs. 6 ▸(*a*)–6 ▸(*c*)]. For the RNA structure, the *R*_work_ and the *R*_free_ values become significantly worse compared with the other three protein structures. Nevertheless, more than 93% of residues could still be accurately built in the final structure, which largely resembles the reference PDB model [Fig. 6 ▸(*d*)]. More examples of automatic structural determinations based on the identified heavy-atom sites produced by the proposed phase-retrieval algorithm are experimentally validated and the results are further listed in Table S3 of the supporting information. Overall, we have experimentally demonstrated that automatic *de novo* macromolecular structure determination is possible on the basis of the modified phase-retrieval algorithm.

## 4. Discussion and conclusions

This series of tests demonstrated that the modified phase-retrieval algorithm exhibits remarkable robustness and versatility for SAD substructure determination. This is primarily evident in the following ways: (i) by introducing the π-half phase perturbation and the tangent formula, the standard RAAR algorithm significantly accelerates its convergence to the accurate solution while simultaneously improving both the accuracy and the chance of success for SAD substructure determination; (ii) the algorithm presented in this study is capable of solving substructures from a variety of SAD datasets containing a range of heavy-atom types (such as Se, S, halogens and metals) for diverse macromolecular structures, including proteins and nucleic acids; (iii) even for the challenging native-SAD datasets with relatively weak anomalous signals, the algorithm still works and maintains a similar performance.

In this work, we have experimentally demonstrated that the success of substructure determination is largely dependent on the strength of anomalous signals and the accuracy is likely to be associated with truncated anomalous resolution. However, this assumption does not always hold true. For example, it was observed that the dataset for the PDB entry 3fki (Meyer *et al.*, 2009 ▸) can be successfully phased even though the anomalous resolution is truncated to a limit value of 6.72 Å. Intriguingly, for the native-SAD dataset with a very weak anomalous signal of 7.92σ (PDB entry 6s1d), all 9 anomalous peaks originating from sulfur atoms could still be accurately located. Of particular note, under native-SAD situations, it inevitably poses the challenge to identify all possible S atoms in the presence of super-sulfurs (Debreczeni *et al.*, 2003 ▸). In most cases, we are only able to find the positions of super-sulfurs instead of individual S peaks, possibly due to the truncated anomalous resolution and the approach used to search for peaks. For instance, the dataset for PDB entry 6o8a (Guo *et al.*, 2019 ▸) contains 8 super-sulfurs and 1 sulfur atom, yet we are only able to determine the precise positions of five super-sulfurs and one sulfur atom, failing to identify all coordinates of both S–S peaks.

Note that the modified phase-retrieval algorithm is flexible in the requirement for an exact estimate of the number of substructure atoms. This parameter, if input, only serves to determine the number of peaks that will be extracted from the difference electron-density map. In essence, the phase-retrieval algorithm is a truly *ab initio* phasing method, functioning independently of any prior knowledge of biological or chemical composition. In addition, the parameterization of the algorithm is very simple and can be self-adjusted according to each specific dataset. More importantly, the modified phase-retrieval algorithm can be seamlessly interfaced with the current widely used programs for automatic structure solution, thus paving the way for its convenient usage or integration into other macromolecular structure solution pipelines.

Future work will focus on exploring potential improvements of the proposed algorithm by optimizing the framework of the modified phase-retrieval algorithm or combining other powerful approaches, such as better starting phases consistent with the Patterson function and a more accurate peak-search strategy. It is hoped that our new procedure can provide an alternative route to SAD substructure determination, particularly under the most challenging native-SAD conditions.

## 5. Algorithm availability

The modified phase-retrieval algorithm is written in standard Fortran90 based on the Linux operating system, and requires an FFTW3 library for the fast Fourier transform [https://www.fftw.org (Frigo & Johnson, 2005 ▸)], the *CCP4* subroutine libraries for basic crystallographic operations (Collaborative Computational Project, 1994 ▸) and fgsl/gsl for random number generation [https://www.gnu.org/software/gsl/ (Galassi *et al.*, 2002 ▸)]. The *CCP4* version used in the test is 8.0.012 (Winn *et al.*, 2011 ▸). The source code is freely available at https://github.com/fuxingke0601/the-modified-phase-retrieval-algorithm. The electron-density maps and structures in Figs. 3 ▸ and 6 ▸ were prepared using *PYMOL* (https://pymol.org/).

## 6. Related literature

The following reference is cited in the supporting information: Uervirojnangkoorn *et al.* (2013 ▸).

## Supplementary Material

Supporting figures and tables. DOI: 10.1107/S2052252524004846/it5034sup1.pdf

## Figures and Tables

**Table 1:** Peak heights of two structures with PDB entries 6s1d and 6fms obtained from *ANODE*

		Fractional coordinates					
PDB entry	Atom	*x*	*y*	*z*	Height/σ	Distance† (Å)	Nearest residue
6s1d	S1	−0.05688	0.16426	0.34437	11.1	1.024	Cys66
	S2	−0.15186	0.40283	0.26001	10.02	0.453	Cys134
	S3	0.01665	0.31268	0.15566	9.88	0.323	Cys164
	S4	−0.07461	0.22382	0.19489	9.72	0.526	Cys149
	S5	0.03983	0.45661	0.27204	9.4	0.723	Cys126
	S6	0.03077	0.10099	0.31691	9.21	0.994	Cys71
	S7	0.01511	0.39559	0.35027	9.16	0.758	Cys9
	S8	0.35417	0.40445	0.27506	8.64	0.375	Cys121
	S9	0.11437	0.34554	0.29566	8.25	0.457	Met112
6mfs	Se1	0.1915	0.08295	0.34115	12.29	0.353	Mse36
	Se2	−0.08708	0.34002	0.05928	11.86	0.125	Mse152
	Se3	0.06833	0.34298	0.39885	11.72	0.328	Mse152
	Se4	0.10187	−0.22588	0.05925	10.99	0.354	Mse152
	Se5	0.1915	0.06116	0.15012	9.95	1.006	Mse36
	Se6	0.15543	0.20962	0.17051	9.9	0.197	Mse117
	Se7	−0.13067	−0.21633	0.46882	9.57	0.256	Mse152
	Se8	0.17528	−0.07826	0.31107	9.5	0.172	Mse117
	Se9	−0.19777	0.08121	0.14722	7.97	0.422	Mse36
	Se10	−0.20254	0.07335	0.35372	7.74	0.562	Mse36
	Se11	−0.15011	0.22928	0.30583	6.23	0.553	Mse117
	Se12	0.0344	0.35134	0.1398	4.03	1.632	Mse157

† The distance between one anomalous peak and its nearest heavy-atom site from the corresponding PDB structure.

**Table 2:** Results of four representative macromolecular structures successfully determined using the *IPCAS* pipeline with the identified heavy-atom sites solved by the modified phase-retrieval algorithm as input

PDB entry	Type	*n* sites†	Programs‡	FOM	*R*_work_/*R*_free_	Completeness	Accuracy	R.m.s.d.§ (Å)	Run time	
									Phase retrieval for each trial (s)	*IPCAS* for each cycle (min)
4qk0	Protein	56/63 Se	O + D + P/B	0.387	0.213/0.251	2405/2484 (96.82%)	2390/2484 (96.22%)	0.26	28	117
3s2s	Protein	4/4 Zn + 4/4 As	O + D + P/B	0.351	0.216/0.236	721/726 (99.31%)	716/726 (98.62%)	0.23	71	48
3fys	Protein	9/10 S	O + D + P/B	0.376	0.202/0.256	275/282 (97.52%)	276/282 (97.87%)	0.23	9	35
5ndi	RNA	4/4 Br	O + D + B/P	0.401	0.279/0.311	63/76 (78.95%)	71/76 (93.42)	0.24	15	44

† Number of sites found in the asymmetric unit (a.u.) compared with the published values.

‡ Programs used in the cycle of model extension iterations in *IPCAS* (alternate mode). Program codes: O = *OASIS*, D = *DM*, B = *Buccaneer*, P = *Phenix.AutoBuild* (quick mode).

§ Root mean square deviations of the Cα positions after structural alignment against the final PDB structures.
