# Supplementary material for: A modified phase-retrieval algorithm to facilitate automatic de novo macromolecular structure determination in single-wavelength anomalous diffraction
Source: IUCrJ. 2024 Jun 21;11(Pt 4):587–601. doi: 10.1107/S2052252524004846 (PMC11220887; doi:10.1107/S2052252524004846)
Supplement: Supplementary file 1 [file m-11-00587-sup1.pdf]

IUCrJ

Volume 11 (2024)

Supporting information for article:

A modified phase-retrieval algorithm to facilitate automatic *de novo* macromolecular structure determination in single-wavelength anomalous diffraction

Xingke Fu, Zhi Geng, Zhichao Jiao and Wei Ding

S1. The criterion for successful substructure solution

As shown in Figure S1, we evaluated three different figures of merit: electron density skewness (Uervirojnangkoorn et al., 2013), Rfactor and the Pearson correlation coefficient (CC). Electron density skewness is an inadequate criterion, as it does not effectively differentiate between successful and failed trials. However, both the CC and Rfactor are able to indicate a successful trial through a distinct jump in their values when converging to the correct solution. But CC values show a strikingly more pronounced differentiation between solutions and non-solutions (1.26 times) compared to Rfactor (0.11 times), so the CC is used as a criterion to indicate whether the trial has been converged to correct solution in the testing of this work.

The Rfactor is calculated using the formula:

$$R = \frac{\sum_{hkl} \big| |E_o| - k|E_c| \big|}{\sum_{hkl} |E_o||} \qquad (1)$$

Where Eo and Ec represent the observed and calculated normalized amplitudes, k is the weight. And Ec is derived from the Fourier transform of the electron density map after real-space restraints. The skewness is calculated using the formula:

$$skew = \langle \rho^3 \rangle / \langle \rho^2 \rangle^{\frac{3}{2}} \qquad (2)$$

Where the electron density (ρ) was calculated at all the grid points in the asymmetric unit.

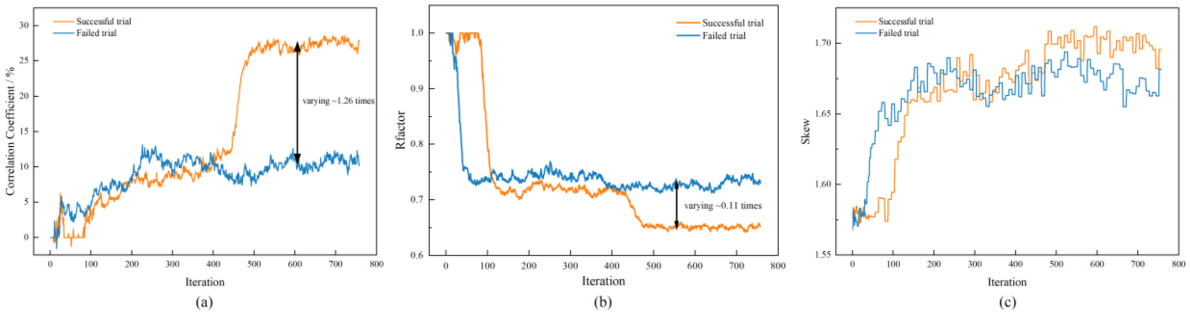

Figure S1 Comparison of the Pearson correlation coefficient in (a), Rfactor in (b) and electron density skewness in (c) for protein with PDB entry 6E9C. Each graph indicates that the runs of the standard RAAR algorithms with the π-half variant across 750 Fourier iterations for successful (in origin) and failed (in blue) trials.

S2. The parameter w_{best} of the modified phase-retrieval algorithm

An essential enhancement to the modified phase-retrieval algorithm is the π-half phase perturbation for weak reflections, that involves perturbing a percentage of reflections considered to be weak based on their experimental amplitudes. The critical consideration is that how to determinate an optimal percentage (w_{best}) of weak reflections for each data set. In 2008, Oszlányi and Sütő

summarized that the fraction of reflections considered weak was typically ranged from ω = 10% to 50% of all data within the resolution sphere. And they suggested that testing ω values of 20% or 40% for each data set is generally sufficient (Oszlányi & Sütő, 2008).

But, In the realm of substructure determination, we found the optimal percentage (w_{best}) typically falls within the range of 20% to 50%. When ω exceeds a certain threshold (w_{limit}), the modified phase-retrieval algorithm is prone to diverge, resulting in the Pearson correlation coefficient (CC) value being 'Not a Number (NaN)' due to excessive perturbations, preventing the algorithm to stabilize at minima. Notably, for certain data sets, only a narrow range of ω yields converged results. So, we have developed a novel method for estimating the percentage (w_{best}). Through our parameter ω testing, we revealed a consistent trend indicating that the optimal percentage (w_{best}) tends to be slightly lower than the value of w_{limit} that triggers the algorithm to approach divergence (as shown in Fig. S2). In the process of implementing the algorithm, we conduct tests with ω values of 0.2, 0.3, 0.4 and 0.5, each subjected to 10 trials to determine an approximate value for w_{limit}, followed by the estimation of the optimal percentage (w_{best}). And the method has been scripted into a bash script to automatically estimate w_{best}. The results obtained from the SAD experimental data sets in this work have demonstrated the appropriateness of this strategy for substructure determination.

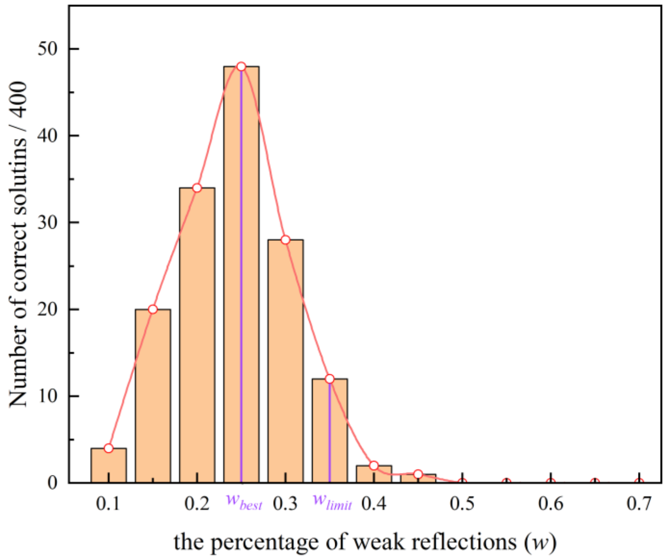

Figure S2 Number of correct (converging) solutions in 400 trials with different percentage (w) of weak reflections for protein with PDB entry 4YF1. $w_{best} = 0.25$ and $w_{limit} = 0.35$. When w exceed w_{limit}, a large number of trials diverged, leading to 'NaN' values for the corresponding CC. And the optimal percentage (w_{best}) is a little lower than w_{limit}.

S3. The relationship between positional difference and the truncated anomalous resolution

We analysis the relationship between positional difference and the truncated anomalous resolution. As shown in Fig. S3, for the mean error and r.m.s.d., we found that the values of positional

difference were positively related to the truncated anomalous resolution. The linear fitting parameters are shown in Table S1. This suggested that lower resolution in truncated data could leads to increased positional uncertainty. However, it's important to note that the low truncated anomalous resolution often results from the poor quality of the raw experimental data. Additionally, the positional difference may also be influenced by peak-seeking method, algorithm parameters, and other factors.

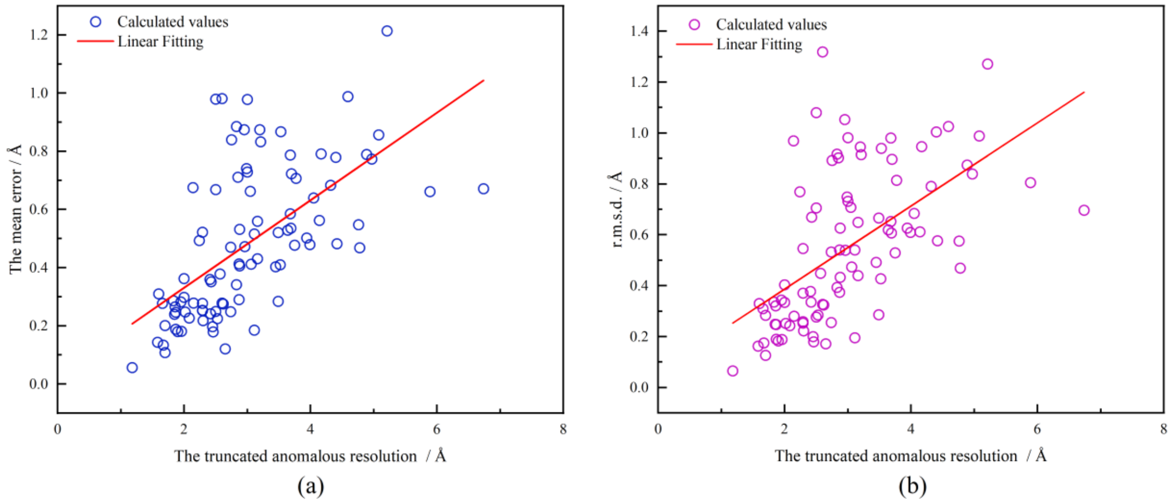

Figure S3 Correlation between positional difference and the truncated anomalous resolution for the 93 SAD data sets successfully solved by the modified phase-retrieval algorithm. Each circle represents a single data set. (a) The mean error of calculated heavy-atom coordinates and corresponding fitting curve. (b) r.m.s.d. and corresponding fitting curve.

Table S1 Extracted fitting parameters obtained from the positional difference of calculated heavy-atom substructures. The equation of linear fitting is $y = a * x + b$.

	Fitting parameters	
	Slope (a)	Intercept (b)
the mean error in Fig S2(a)	0.150521	0.0294895
r.m.s.d. in Fig S2(b)	0.163337	0.0598711

S4. Comparison of the modified phase-retrieval algorithm and the standard RAAR algorithm

We compare the performance of the modified phase-retrieval algorithm and the standard RAAR algorithm in terms of substructure determination. As shown in Fig. S4, the results can be categorized into three groups: firstly, 72 data sets can be solved by both algorithms; secondly, for 7 data sets located in the bottom left corner of Fig. S4, neither one is effective; thirdly, for the remaining 21 data

sets in the upper left corner, the modified phase-retrieval algorithm successfully solve the heavy-atom substructures while the standard RAAR algorithm does not.

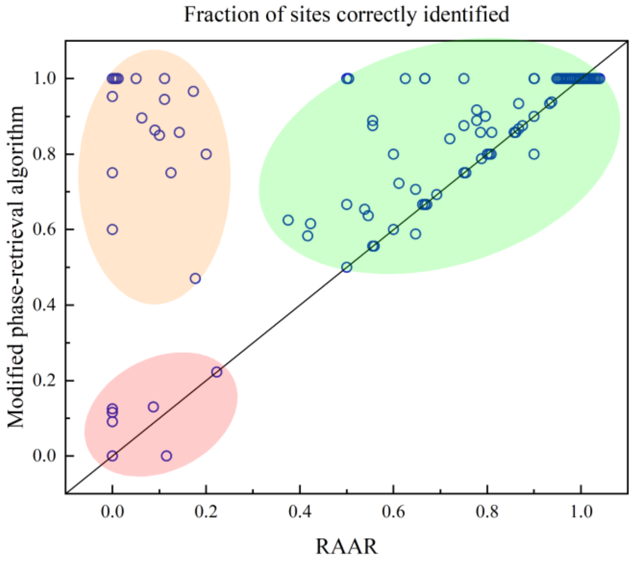

Figure S4 Fraction of substructure correctly determined by the modified phase-retrieval algorithm and the standard RAAR algorithm for 100 SAD data sets. Each circle in the graph represents a single date set. The horizontal coordinates of the overlapping data points are artificially adjusted by an increase or decrease of 0.004.

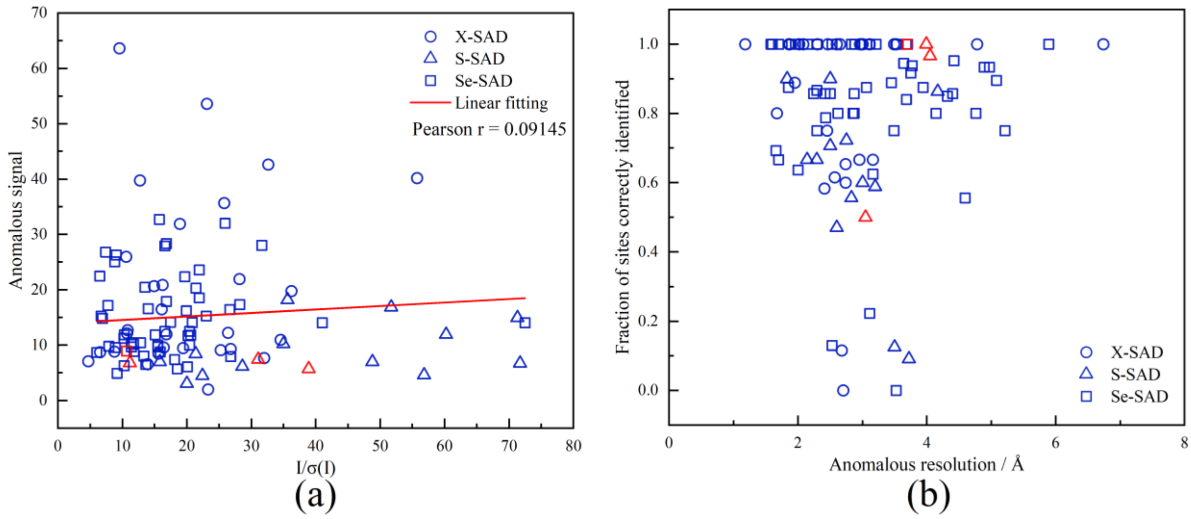

Figure S5 (a) The relationship between the anomalous signal and signal-to-noise ratio for 100 SAD datasets. The red line is the line of best linear fit with a Pearson’ r of 0.09145. (b) The fraction of heavy-atom sites correctly identified as a function of the truncated anomalous resolution. Each symbol in the graph represents a single date set. The circle, triangle and square represent X-SAD data set (X represents iodine, bromine, or metal ions), S-SAD data set and Se-SAD data set, respectively. The substructure searches carried out with default parameters are shown in blue and the red ones

indicate substructures determined with failure initially but that could be solved by further adjustment of some parameters.

S5. 100 sets of SAD experimental data

A total of 100 SAD experimental data sets are randomly selected from PDB, encompassing diverse testing data types. These sets can be categorized into 88 protein sets and 12 nucleic acid sets based on macromolecular type, and 55 Se-SAD sets, 16 S-SAD sets, and 29 X-SAD sets (where X represents iodine, bromine, or metal ions) based on heavy-atom type. The crystallographic data encompass a wide range of resolutions (1.1-3.9 Å), the number of sites in the asymmetric unit (1-63), and seven crystallographic systems (triclinic, monoclinic, orthorhombic, tetragonal, trigonal, hexagonal, and cubic)

Table S2 Experimental data and results of heavy-atom substructure determination using the modified phase-retrieval algorithm.

[1]RES$_{ref}$: experimental diffraction resolution; [2] Bijvoet ratio: the mean ratio of Bijvoet difference to the total experimental anplitude; [3]a.s. with *ANODE*: the anomalous signal from heavy-atom substructure is calculated with *ANODE*; [4] a.s. with *FFT*: the anomalous signal from heavy-atom substructure is calculated with *FFT*; [5]nsites: the number of sites found in the asymmetric unit (a.u.) compared with published values; [6]n.c.t.: the number of converging (correct) trials out of 400. [7]error: the mean error of calculated heavy-atom substructure without *BP3* refinement; [8]r.m.s.d.: the root mean square deviation of calculated heavy-atom substructure without *BP3* refinement; [9]error with BP3: the mean error with *BP3* refinement; [10]r.m.s.d. with BP3: r.m.s.d. with *BP3* refinement. Number 90-93 (7TL5, 6I59, 6S1D, 4WBX), the substructures could be determined using the modified phase-retrieval algorithm by adjusting parameters. Number 94-100 (7TRV, 7THW, 7TB5, 5WTI, 7B76, 4TKQ, 5FQ5), the determination of substructures is unsuccessful.

No.	PDB entry	Experimental information							Results					
		Type	Space group	[1]RES$_{ref}$ / Å	Heavy atom	[2]Bijvoet ratio	[3]a.s. with *ANODE* / σ	[4]a.s. with *FFT* / σ	[5]nsites	[6]n.c.t.	[7]error / Å	[8]r.m.s.d. / Å	[9]error with *BP3* / Å	[10]r.m.s.d. with *BP3* / Å
1	3K9G	protein	$P\,4_3\,2_1\,2$	2.25	I	5.47	15.17	10.95	7/12	5	0.359	0.377	0.125	0.128
2	4QK0	protein	$P\,1\,2_1\,1$	2.258	Se	9.18	19.97	15.27	56/63	30	0.403	0.492	0.243	0.362
3	5C82	protein	$C\,1\,2\,1$	2.2	Se	10.04	25.08	20.31	4/4	66	0.225	0.285	0.144	0.186
4	8I59	protein	$P\,4_1\,2_1\,2$	3.205	Se	6.85	16.3	11.69	12/14	3	0.779	1.004	0.336	0.38
5	5JD2	protein	$P\,1\,2_1\,1$	1.319	Se	7.4	28.85	16.61	3/4	38	0.253	0.259	0.29	0.309
6	5OQ2	protein	$P\,1\,2_1\,1$	2.3	Se	6.55	23.15	17.33	5/8	16	0.431	0.44	0.176	0.197
7	7E1D	protein	$P\,2_1\,2_1\,2_1$	2.001	Se	8.22	21.27	16.41	4/4	51	0.362	0.404	0.477	0.54
8	7QOC	protein	$P\,6_3$	2.299	Se	6.42	16.5	11.88	8/10	100	0.413	0.541	0.465	0.606
9	5HHK	protein	$P\,1\,2_1\,1$	1.398	Se	11.26	25.11	20.46	26/33	75	0.351	0.67	0.211	0.444
10	6CKN	protein	$P\,4_3\,2_1\,2$	2.49	Se	4.64	19.84	11.83	2/2	22	0.185	0.195	0.146	0.183

11	4ZE9	protein	$C\,2\,2\,2_1$	2.645	Se	9.11	11.03	8.84	17/18	3	0.529	0.62	0.529	0.62
12	5AUJ	protein	$P\,6_3$	2.498	Se	2.31	11.67	7.4	6/7	27	0.668	0.706	0.463	0.408
13	5IQY	protein	$C\,2\,2\,2_1$	2.4	I	8.48	10.76	7.68	16/26	6	0.378	0.449	0.276	0.323
14	3FKI	protein	$C\,2\,2\,2_1$	3.878	Zn	14.18	17.28	6.58	8/8	3	0.671	0.697	1.056	1.12
15	3O2E	protein	$P\,4_2\,2_1\,2$	1.95	I	7.59	13.21	9.47	8/9	17	0.282	0.343	0.198	0.228
16	4US7	protein	$I\,2\,2\,2$	1.96	S	1.8	22.38	16.85	3/5	4	0.978	0.982	0.41	0.436
17	4XVZ	protein	$C\,1\,2\,1$	2.49	Se	10.96	13.25	8.05	17/20	41	0.683	0.791	0.431	0.493
18	4YF1	protein	$P\,1\,2_1\,1$	1.85	Se	7.53	33.58	28.06	8/8	42	0.342	0.393	0.175	0.256
19	5IRR	protein	$C\,1\,2\,1$	2.04	Se	8.34	17.09	9.67	8/10	5	0.562	0.612	0.265	0.331
20	5KJH	protein	$P\,1\,2_1\,1$	2.268	Zn	4.11	26.42	21.95	8/8	27	0.516	0.541	0.208	0.231
21	5LG6	protein	$P\,1\,2_1\,1$	2.5	Se	11.27	13.11	8.66	43/48	2	0.856	0.989	0.374	0.536
22	5LLW	protein	$P\,2_1\,2_1\,2_1$	2.801	Se	9.48	13.52	8.77	33/36	8	0.477	0.529	0.402	0.683
23	5LOI	protein	$I\,4\,2\,2$	3.153	Se	7.16	17.76	15.24	7/8	40	0.502	0.626	0.604	0.883
24	3S2S	protein	$P\,2_1\,2_1\,2_1$	1.7	Zn, As	3.55	51.33	42.61	8/8	29	0.278	0.327	0.086	0.096
25	6QE4	protein	$P\,1$	2.3	I	14.57	11.64	8.75	8/12	5	0.559	0.649	0.498	0.575
26	6E9C	protein	$P\,2_1\,2_1\,2_1$	3.2	Se	6.65	9.16	6.29	14/15	55	0.789	0.874	0.595	0.658
27	5FGX	protein	$P\,4_1\,2_1\,2$	2.134	S	3.49	17.53	14.94	12/18	11	0.675	0.969	0.369	0.504
28	6H2M	protein	$I\,2\,2\,2$	1.929	Mn, Ca	5.76	39.1	31.93	2/2	5	0.121	0.172	0.021	0.029
29	3FYS	protein	$P\,1\,2_1\,1$	2.5	S	3.48	17.11	11.9	9/10	131	0.25	0.277	0.207	0.254
30	7L84	protein	$P\,4_3\,2_1\,2$	1.704	S	4.05	18.8	18.16	9/10	2	0.286	0.336	0.066	0.069
31	5TCL	protein	$P\,4_1\,2_1\,2$	3.201	S	14.23	9.13	8.4	10/17	9	0.874	0.946	0.859	0.895
32	5CW1	protein	$P\,4_3\,2_1\,2$	1.45	I	3.96	42.77	40.17	4/5	47	0.134	0.175	0.088	0.119
33	8ENA	protein	$P\,4_1\,2_1\,2$	2.501	S	7.72	9.15	3.08	12/17	9	0.979	1.08	0.677	0.733
34	3FBX	protein	$C\,1\,2\,1$	2.401	S	3.43	12.42	7	19/22	2	0.791	0.947	0.455	0.482
35	4TNO	protein	$P\,4_1\,2_1\,2$	2.134	S	2.14	8.97	6.73	2/3	16	0.522	0.546	0.358	0.383
36	5XFD	protein	$P\,6_5$	1.5	Se	9.44	29.05	22.48	2/2	2	0.252	0.253	0.203	0.225
37	5CXR	protein	$C\,2\,2\,2_1$	2.001	Br	4.9	17.29	12.22	3/5	8	0.249	0.255	0.171	0.19
38	5T3L	DNA	$P\,2_1\,2_1\,2_1$	1.579	Se	7.15	27.56	23.58	2/2	50	0.144	0.162	0.094	0.107
39	6HF7	protein	$P\,4\,2_1\,2$	1.96	Tb	12.99	59.42	53.62	3/4	90	0.197	0.2	0.063	0.079
40	5T3G	protein	$P\,4_1\,2_1\,2$	1.554	Se	4.17	21.46	14.05	9/13	17	0.277	0.31	0.25	0.27
41	6YSO	protein	$P\,1\,2_1\,1$	3.13	V	7.13	40.36	20.69	2/2	56	0.468	0.469	0.262	0.267
42	6DTZ	protein	$P\,2_1\,2_1\,2_1$	1.36	Fe	8.38	41.61	39.78	4/4	23	0.188	0.19	0.044	0.051
43	6JJF	DNA	$C\,1\,2\,1$	1.47	K	6.92	11.96	9.32	3/3	10	0.248	0.252	0.132	0.14
44	5CKI	DNA	$P\,4_3\,2_1\,2$	2.985	Co	9.6	11.01	9.11	4/4	65	0.741	0.749	0.723	0.73
45	5U31	DNA, protein	$I\,2\,2\,2$	2.89	Se	11.43	15.1	10.19	20/21	2	0.482	0.577	0.394	0.484
46	6JOO	DNA, RNA, protein	$C\,1\,2\,1$	2.9	Se, Zn	7.65	16.557	11.21	14/14	98	0.661	0.805	0.459	0.524
47	5NDI	RNA	$P\,2_1\,2_1\,2_1$	2.57	Br	8.39	17.48	12.14	4/4	56	0.472	0.539	0.831	0.852

48	5NDH	RNA	$P\,2_1\,2_1\,2_1$	1.81	Br	8.98	27.75	20.88	4/4	53	0.227	0.243	0.281	0.3
49	7ELP	RNA	$P\,2_1\,2_1\,2$	2.788	Ir	13.33	19.33	7.09	3/3	10	0.284	0.286	0.271	0.287
50	6TFE	RNA	$I\,2\,2\,2$	2.3	Br	4.03	19.42	16.49	2/2	14	0.41	0.427	0.523	0.553
51	3LOA	RNA	$P\,6_4$	2.298	Br	4.97	24.81	19.76	2/2	390	0.218	0.223	0.218	0.223
52	4j1G	protein	$P\,1$	3.07	Se	8.35	9.64	7.95	20/20	41	0.867	0.94	0.891	0.999
53	2XSK	protein	$C\,1\,2\,1$	1.699	Se	7.06	21.75	17.91	2/3	10	0.201	0.284	0.168	0.169
54	5D9D	protein	$P\,2_1\,2_1\,2_1$	1.7	Hg	7.03	29.43	25.96	2/3	5	0.874	1.053	0.094	0.094
55	6FMS	protein	$C\,1\,2\,1$	3	Se	11.52	7	4.92	12/16	18	1.214	1.271	0.576	0.659
56	6JGF	protein	$P\,3_1\,2\,1$	2.147	Se	31.03	30.3	14.04	2/2	3	0.278	0.28	0.053	0.055
57	6FMR	protein	$C\,2\,2\,2_1$	2.7	Se	14.59	11.11	9.59	18/18	6	0.723	0.897	0.384	0.456
58	7O51	protein	$P\,4_1\,2_1\,2$	2.2	S	3.8	9.65	6.98	13/18	5	0.84	0.892	0.788	0.872
59	7TEM	protein	$P\,2_1\,2_1\,2_1$	1.645	Se	11.03	37.35	32.03	18/21	106	0.29	0.374	0.129	0.247
60	7ENM	protein	$P\,2_1\,2_1\,2_1$	1.696	Se	16.15	28.41	25.1	3/3	43	0.108	0.126	0.106	0.136
61	7RB4	protein	$P\,2_1\,2_1\,2_1$	2.185	I	7.66	13.3	9.58	17/26	25	0.47	0.532	0.347	0.42
62	7RFO	protein	$P\,4_1\,2_1\,2$	3.016	Se	7.19	15.25	10.03	15/16	2	0.707	0.814	0.707	0.814
63	6ZPP	protein	$P\,1\,2_1\,1$	1.5	Se	16.68	28.79	26.82	3/3	64	0.31	0.33	0.169	0.18
64	7NBV	protein	$P\,2_1\,3$	1.87	Br	7.86	26.61	11.95	1/1	10	0.247	0.247	0.247	0.247
65	7TJ1	protein	$P\,2_1\,2_1\,2_1$	2.1	Se	10.87	17.94	12.52	16/16	12	0.406	0.433	0.19	0.234
66	7WBK	protein	$C\,1\,2\,1$	2.74	Se	7.29	19.28	14.14	8/10	50	0.548	0.576	0.366	0.401
67	7TBS	protein	$I\,2\,2\,2$	1.958	Se	10.86	34.23	27.92	3/3	17	0.181	0.189	0.087	0.101
68	7TMU	protein	$P\,1\,2_1\,1$	2.544	Se	10.22	14.35	12.52	9/12	107	0.521	0.666	0.482	0.69
69	7RR3	protein	$P\,2_1\,2_1\,2_1$	2.24	Se	14.73	13.75	10.43	6/7	14	0.493	0.769	0.357	0.502
70	7TFQ	protein	$P\,2_1\,2_1\,2$	1.744	Se	6.95	19.08	14.11	7/11	9	0.298	0.335	0.256	0.268
71	7TWC	protein	$C\,1\,2\,1$	1.847	Se	10.47	34.09	28.34	7/8	11	0.24	0.248	0.124	0.188
72	7V1Q	protein	$P\,1\,2_1\,1$	1.581	Se	14.1	31.85	32.69	24/24	25	0.833	0.915	0.299	0.393
73	7DNT	protein	$P\,2_1\,2_1\,2$	2.499	Se	12.81	12.61	9.78	9/9	23	0.532	0.627	0.555	0.623
74	7TCB	protein	$P\,1\,2_1\,1$	2.678	Se	12.29	11.77	9.71	21/25	6	0.787	0.981	1.029	1.169
75	7V3B	protein	$P\,2_1\,2_1\,2_1$	1.795	Hg	22.31	85.69	63.64	1/1	23	0.179	0.179	0.192	0.192
76	7V38	protein	$P\,2_1\,2_1\,2_1$	2.403	Hg	5.39	15.1	12.7	2/2	31	0.729	0.732	0.556	0.557
77	7BLL	protein	$P\,1\,2\,1$	1.762	Se	14.81	33.05	10	18/21	39	0.242	0.336	0.164	0.244
78	7MSN	protein	$P\,4_3\,2_1\,2$	3.002	Se	6.53	9.2	6.08	10/18	1	0.988	1.026	0.513	0.614
79	7E6V	protein	$P\,1$	1.831	Se	16.02	19.44	17.17	13/15	339	0.277	0.371	0.277	0.371
80	7T8L	protein	$P\,1\,2_1\,1$	2	Se	11.5	17.22	14.82	14/15	27	0.773	0.839	0.306	0.358
81	7DSU	protein	$P\,2_1\,2_1\,2_1$	3.2	Se	13.05	15.44	10.44	14/14	10	0.585	0.652	0.488	0.577
82	7PTB	protein	$P\,6_1$	2.082	Se	6.37	31.43	22.37	4/5	300	0.711	0.902	0.513	0.608
83	7AOV	protein	$P\,1\,2_1\,1$	2	Se	14.64	16.94	11.86	14/16	67	0.412	0.474	0.406	0.474
84	6JJH	protein	$P\,1\,2\,1$	1.74	K	3.94	10.85	8.46	3/3	101	0.265	0.321	0.311	0.383
85	6S19	protein	$P\,4_1\,2_1\,2$	2.65	S	5.96	12.01	10.22	10/18	7	0.885	0.917	0.513	0.614

86	2OBZ	DNA	$P\,2_1\,2_1\,2_1$	1.1	Br	10.07	30.19	35.69	2/2	102	0.056	0.065	0.051	0.054
87	6O8A	protein	$P\,4_1\,2_1\,2$	2.501	S	5.82	10.45	4.49	8/17	2	0.981	1.319	0.759	0.825
88	7F9H	protein	$C\,1\,2\,1$	1.777	Se	12.11	10.58	26.27	4/4	35	0.18	0.183	0.084	0.088
89	7TRW	protein	$P\,6_2$	2.28	Se	4.35	10.79	18.54	4/5	164	0.275	0.324	0.311	0.338
90	7TL5	protein	$P\,2_1\,2_1\,2_1$	2.688	Se	8.72	7.92	8.97	40/40	3	0.536	0.607	0.383	0.44
91	6I59	protein	$P\,2_1\,2_1\,2$	2.95	S	3.41	10.52	7.39	28/29	2	0.64	0.684	0.396	0.46
92	6S1D	protein	$P\,4_1\,2_1\,2$	2.65	S	6.45	39.09	6.75	9/18	1	0.662	0.708	0.749	0.75
93	4WBX	protein	$F\,2\,2\,2$	2.301	S	13.31	22.93	5.69	10/10	15	0.479	0.61	0.483	0.567
94	7TRV	protein	$P\,1\,2_1\,1$	1.795	Se	14.59	7.97	6.52	2/9	0	-	-	-	-
95	7THW	protein	$P\,1\,2_1\,1$	2.199	Se	18.05	7	5.73	3/23	0	-	-	-	-
96	7TB5	protein	$P\,6_1\,2\,2$	2.296	Se	4.32	23.17	16.19	0/4	0	-	-	-	-
97	5WTI	DNA, RNA, protein	$C\,1\,2\,1$	2.682	Mg	5.15	2.55	2.01	3/26	0	-	-	-	-
98	7B76	protein	$P\,6_1\,2\,2$	2.698	I	8.42	13.57	8.83	0/3	0	-	-	-	-
99	4TKQ	protein	$P\,6_5\,2\,2$	2.801	S	3.48	8.76	4.59	1/8	0	-	-	-	-
100	5FQ5	protein	$C\,1\,2\,1$	2.136	S	4.18	10.9	6.15	2/22	0	-	-	-	-

Table S3 A list of some successfully determined SAD macromolecular structures using the *IPCAS* pipeline with the identified heavy-atom sites solved by the modified phase-retrieval algorithm as input.

PDB entry	type	[1]nsites	[2]Programs	FOM	R_{work}/R_{free}	Completeness	Accuracy	[3]r.m.s.d.	Run time	
									Phase retrieval for each trial (s)	*IPCAS* for each cycle (min)
2XSK	protein	2/3 Se	O+D+P/B	0.41	0.274/0.290	85/93 (91.40%)	87/93 (93.55)	0.31	26	20
3FBX	protein	19/22 S	O+D+P/B	0.399	0.217/0.245	485/519 (93.45%)	507/519 (97.69)	0.25	21	112
3K9G	protein	7/12 I	O+D+P/B	0.42	0.266/0.316	221/226 (97.79%)	219/226 (96.90%)	0.26	37	36
3O2E	protein	8/9 I	O+D+P/B	0.472	0.326/0.345	47/86 (54.65%)	76/86 (88.37)	0.33	17	20
4US7	protein	3/5 S	O+D+P/B	0.31	0.249/0.273	161/178 (90.45%)	171/178 (96.07)	0.16	17	56
4YF1	protein	8/8 Se	O+D+P/B	0.347	0.236/0.258	596/603 (98.84%)	576/603 (95.52%)	0.34	25	104
5C82	protein	4/4 Se	O+D+P/B	0.419	0.206/0.250	152/166 (91.57%)	160/166 (96.39%)	0.43	6	32
5CW1	protein	4/5 I	O+D+P/B	0.363	0.203/0.215	277/279 (99.28%)	277/279 (99.28%)	0.05	80	64
5CXR	protein	3/5 Br	O+D+P/B	0.378	0.247/0.270	185/201 (92.04%)	187/201 (93.03%)	0.33	17	40
5D9D	protein	2/3 Hg	O+D+P/B	0.488	0.221/0.249	282/306 (95.42%)	293/306 (95.75%)	0.18	13	44

5FGX	protein	12/18 S	O+D+P/B	0.305	0.188/0.224	202/207 (97.58%)	202/207 (97.58%)	0.12	15	40
5HHK	protein	26/33 Se	O+D+P/B	0.398	0.212/0.237	504/557 (90.48%)	508/557 (91.20%)	0.45	25	148
5IQY	protein	16/26 I	O+D+P/B	0.458	0.300/0.350	211/211 (100%)	205/211 (97.16%)	0.42	29	32
5LG6	protein	43/48 Se	O+D+P/B	0.181	0.213/0.258	1584/1728 (91.67%)	1591/1728 (92.07%)	0.39	12	232
5LOI	protein	7/8 Se	O+D+P/B	0.477	0.318/0.368	298/383 (77.81%)	298/383 (77.81%)	0.91	79	56
5NDH	RNA	4/4 Br	O+D+B/P	0.4	0.359/0.385	39/56 (69.64%)	41/56 (73.21%)	0.18	36	136
5T3G	protein	9/13 Se	O+D+P/B	0.371	0.193/0.211	205/206 (99.51%)	205/206 (99.51%)	0.05	86	76
6CKN	protein	2/2 Se	O+D+P/B	0.413	0.294/0.333	114/130 (87.69%)	120/130 (92.31%)	0.27	9	32
6DTZ	protein	4/4 Fe	O+D+P/B	0.345	0.276/0.295	177/183 (96.72%)	177/183 (96.72%)	0.07	33	76
6HF7	protein	3/4 Tb	O+D+P/B	0.42	0.228/0.254	569/573 (99.30%)	558/573 (97.38%)	0.29	89	160
6JGF	protein	2/2 Se	O+D+P/B	0.409	0.399/0.409	71/115 (61.74%)	86/115 (74.78%)	0.44	11	28
6TFE	RNA	2/2 Br	O+D+B/P	0.386	0.397/0.427	30/50 (60%)	38/50 (76%)	0.31	12	28
6ZPP	protein	3/3 Se	O+D+P/B	0.385	0.243/0.279	136/154 (88.31%)	141/154 (91.56%)	0.29	14	32
7AOV	protein	14/16 Se	O+D+P/B	0.398	0.229/0.252	728/738 (98.64%)	725/738 (98.24%)	0.32	18	120
7BLL	protein	18/21 Se	O+D+P/B	0.218	0.261/0.281	590/591 (99.83%)	586/591 (99.15%)	0.42	42	40
7E1D	protein	4/4 Se	O+D+P/B	0.5	0.251/0.284	141/185 (76.22%)	175/185 (94.59%)	0.19	28	32
7E6V	protein	13/15 Se	O+D+P/B	0.5	0.243/0.288	327/393 (83.21%)	326/393 (82.95%)	0.51	7	60
7ENM	protein	3/3 Se	O+D+P/B	0.495	0.215/0.248	97/98 (98.98%)	97/98 (98.98%)	0.11	20	28
7F9H	protein	4/4 Se	O+D+P/B	0.359	0.351/0.377	123/182 (67.58%)	119/182 (65.38%)	0.26	87	56
7L84	protein	9/10 S	O+D+P/B	0.424	0.209/0.215	126/129 (97.67%)	126/129 (97.67%)	0.18	38	28
7PTB	protein	4/5 Se	O+D+P/B	0.355	0.242/0.273	313/313 (100%)	313/313 (100%)	0.34	20	92
7QOC	protein	8/10 Se	O+D+P/B	0.5	0.225/0.256	335/372 (90.05%)	331/372 (88.98%)	0.55	32	60
7RB4	protein	17/26 I	O+D+P/B	0.391	0.231/0.273	600/603 (99.50%)	600/603 (99.50%)	0.22	38	72
7TBS	protein	3/3 Se	O+D+P/B	0.496	0.222/0.267	210/218 (96.33%)	211/218 (96.79%)	0.2	56	60
7TEM	protein	18/21 Se	O+D+P/B	0.496	0.203/0.225	775/791 (97.98%)	782/791 (98.86%)	0.16	30	84
7TFQ	protein	7/11 Se	O+D+P/B	0.495	0.216/0.250	272/276 (98.55%)	273/276 (98.91%)	0.12	46	44
7TJ1	protein	16/16 Se	O+D+P/B	0.496	0.213/0.256	499/510 (97.84%)	495/510 (97.06%)	0.19	15	60

7TMU	protein	9/12 Se	O+D+P/B	0.497	0.221/0.268	565/567 (99.65%)	562/567 (99.12%)	0.31	11	92
7TRW	protein	4/5 Se	O+D+P/B	0.499	0.229/0.270	208/208 (100%)	207/208 (99.52%)	0.5	22	60

[1]nsites: the number of sites found in the asymmetric unit (a.u.) compared with published values; [2]program: the programs used in the cycle of model extension iterations in IPCAS (alternate mode). Program codes: O = OASIS, D = DM, B = Buccaneer, P = Phenix.AutoBuild (quick mode). [3]r.m.d.s: the root mean square deviations of Cα positions after structural alignment against the final PDB structures.

References

Oszlányi, G. & Sütő, A. (2008). Acta Crystallographica Section A 64, 123-134.

Uervirojnangkoorn, M., Hilgenfeld, R., Terwilliger, T. C. & Read, R. J. (2013). Acta Cryst. D 69, 2039-2049.
